# Metabolite biomarkers of type 2 diabetes mellitus and pre-diabetes: a systematic review and meta-analysis

**DOI:** 10.1186/s12902-020-00653-x

**Published:** 2020-11-23

**Authors:** Jianglan Long, Zhirui Yang, Long Wang, Yumei Han, Cheng Peng, Can Yan, Dan Yan

**Affiliations:** 1 Beijing Key Laboratory and Joint Laboratory for International Cooperation of Bio-characteristic Profiling for Evaluation of Rational Drug Use, Capital Medical University Affiliated Beijing Shijitan Hospital, Beijing, 100038 China; 2 Chengdu University of Traditional Chinese Medicine, Chengdu, 611130 China; 3 Department of Applied Mathematics and Statistics, Johns Hopkins University, Baltimore, MD 21218 USA; 4 Beijing Physical Examination Center, Beijing, 100077 China; 5 Guangzhou University of Chinese Medicine, Guangzhou, 510006 China

**Keywords:** Metabolite, Biomarker, Type 2 diabetes mellitus, Pre-diabetes; meta-analysis

## Abstract

**Background:**

We aimed to explore metabolite biomarkers that could be used to identify pre-diabetes and type 2 diabetes mellitus (T2DM) using systematic review and meta-analysis.

**Methods:**

Four databases, the Cochrane Library, EMBASE, PubMed and Scopus were selected. A random effect model and a fixed effect model were applied to the results of forest plot analyses to determine the standardized mean difference (SMD) and 95% confidence interval (95% CI) for each metabolite. The SMD for every metabolite was then converted into an odds ratio to create an metabolite biomarker profile.

**Results:**

Twenty-four independent studies reported data from 14,131 healthy individuals and 3499 patients with T2DM, and 14 included studies reported 4844 healthy controls and a total of 2139 pre-diabetes patients. In the serum and plasma of patients with T2DM, compared with the healthy participants, the concentrations of valine, leucine, isoleucine, proline, tyrosine, lysine and glutamate were higher and that of glycine was lower. The concentrations of isoleucine, alanine, proline, glutamate, palmitic acid, 2-aminoadipic acid and lysine were higher and those of glycine, serine, and citrulline were lower in prediabetic patients. Metabolite biomarkers of T2DM and pre-diabetes revealed that the levels of alanine, glutamate and palmitic acid (C16:0) were significantly different in T2DM and pre-diabetes.

**Conclusions:**

Quantified multiple metabolite biomarkers may reflect the different status of pre-diabetes and T2DM, and could provide an important reference for clinical diagnosis and treatment of pre-diabetes and T2DM.

**Supplementary Information:**

The online version contains supplementary material available at 10.1186/s12902-020-00653-x.

## Background

Type 2 diabetes mellitus (T2DM) is a highly prevalent chronic disease that is associated with the development of complications including diabetic retinopathy, kidney disease and diabetic ketoacidosis [1, 2], which represent serious threats to human health. Between 1980 and 2014, the number of adults with diabetes increased from 108 million to 422 million [3], with T2DM accounting for > 90% of these cases [4]. Recent studies have shown that diabetes has become one of the three major diseases in the world with the increasing global prevalence rate [5]. However, the symptoms of T2DM are not very obvious or only partially manifest in the early stages of the disease. Therefore, it is particularly important to identify an early diagnosis and effective treatment for diabetes.

In view of the high incidence of T2DM and its serious consequences, the identification of novel diagnostic markers for T2DM has become a subject of intense research. The existing recognized diagnostic biomarkers of T2DM are blood glucose (including fasting blood glucose and 2 h glucose in oral glucose tolerance test) and hemoglobin A1c. The metabolomic approach aims to identify all the metabolites present in a biologic system, whether cells, tissues or living organisms, to identify their physiologic or pathologic effects [6]. The development of metabolomics makes it possible for metabolites to be identified as biomarkers that may be useful for the diagnosis or treatment of diabetes. For example, amino acids have been proposed to be useful diagnostic biomarkers because the metabolism of amino acids is considerably altered in pre-diabetes and continue to vary over the course of T2DM progression [7, 8]. In particular, tryptophan and branched chain amino acids (BCAAs, including valine, leucine and isoleucine) could represent potentially useful biomarkers of T2DM because their serum concentrations are higher in T2DM patients [9]. Additionally, plasma phospholipid such as phosphatidylinositol and sphingomyelin were capable of discriminating healthy individuals and T2DM patients [10].

It is critical to study of bring data on the appearance of metabolic profile abnormalities before the occurrence of pre-diabetes or T2DM, since this might predict and allow prevent the disease progression to pre-diabetes or T2DM. However, there is no current consensus regarding the use of metabolites as diagnostic biomarkers of T2DM, and part of the results were from clinical single-center or insufficient consideration of mixed factors such as different regions and different populations [11]. Therefore, it is a need for an effective and comprehensive evaluation method for the use of metabolites as diagnostic biomarkers of pre-diabetes or early T2DM. The study from Guasch-Ferré et al. showed that several amino acids were consistently associated with the risk of T2DM [12]. Since then, a number of original studies emerged. We hence undertook a systematic review and meta-analysis of the proposed biomarkers of T2DM or pre-diabetes revealed by published metabolomics and constructed a profile of the metabolite biomarkers. The purpose of this study is to explore metabolite biomarkers integrating biomarkers from different studies through systematic review and meta-analysis, which could provide further evidence for early diagnosis of pre-diabetes and T2DM.

## Methods

The systematic review was conducted according to the Preferred Reporting Items for Systematic Reviews and Meta-Analyses (PRISMA) guidelines [13].

### Data sources and search strategy

The Cochrane Library, EMBASE, PubMed and Scopus were searched for studies published from the earliest available online to May 31, 2019. The search words were “metabonomics”, “metabolomics”, “metabolome”, “type 2 diabetes”, “type 2 diabetics”, “type 2 diabetes mellitus”, “insulin resistance”, “HOMA-IR”, “Impaired glucose tolerance”, “glucose intolerance”, “impaired fasting insulin”, “impaired fasting glucose”, “prediabetic”, “pre-diabetes” and “prediabetes” connected with OR and/or AND. To ensure the relevance of the retrieved results, the “Title, Abstract, Keywords” terms were used in the four databases.

### Study selection and inclusion criteria

The titles, abstracts and full texts of the articles were evaluated after duplicate records were removed. Before literature screening, the inclusion criteria for the publications obtained were formulated by two authors (Long and Yang) as follows: (1) studies conducted in humans; (2) the participants in the study were not gestational diabetes mellitus (GDM), type 1 diabetes mellitus (T1DM) or subjects under 18 years of age; (3) the study included a diabetic group or a prediabetic group and diagnosis was performed according to the international diagnostic guidelines [14]; (4) the article was not a review, conference abstract, editorial or note; (5) the biologic samples analyzed were collected in the fasting state and (6) the study was not conducted with dietary interventions and (or) medications. The publications initially identified as relevant were screened independently by two investigators (Long and Yang) using Endnote X7 (Thomson ResearchSoft, Stanford, USA). If there was any disagreement regarding the selection or inclusion of a study, this was resolved by discussion or by involvement of a third author (Yan). Studies of biomarkers of human pre-diabetes and T2DM identified using metabonomic technology have been included. The prediabetic category included subject who met the above inclusion criteria and had impaired glucose tolerance (IGT) or impaired fasting glucose (IFG) [15].

### Quality assessment and data extraction

The Newcastle-Ottawa Scale (NOS) criteria [16] were used to assess each publication to improve the overall reliability of the extracted data. Three domains, the comparability of cases and controls, selection of cases and controls and exposure, were subdivided into eight risk assessment items. The comparability domain was awarded a maximum of two stars and other items were awarded a maximum of one star, which indicated low, moderate or high risk of bias, respectively. High and low NOS scores reflect low and high risks of bias, respectively.

Two investigators (Long and Yang) independently extracted appropriate information, including the names of the authors and journal, year of publication, study design, population, sample sizes of the case and control groups, the biologic samples obtained, analytic method, determination method, covariates of statistical analysis in the study and the identity and concentrations of the metabolites detected [reported as mean ± standard deviation (SD) or standard error (SE)] in the case and control groups. For the publications that did not provide mean values, we extracted the hazard ratio or odds ratio (OR) and its 95% confidence interval (95% CI). We also extracted the median and interquartile range values from two publications regarding pre-diabetes.

### Statistical analysis

Forest plots for each metabolite for which mean ± SD/SE values were available were produced using Review Manager 5.3 software. The raw data for each metabolite were described in the forest plots, which reflected the weighted contribution of each study. The heterogeneities of the pooled means generated using the forest plots were assessed using the *I*^2^ statistic. For continuous variables, random effect models [17] were used to assess the pooled means when *I*^2^ > 50%; otherwise, fixed effect models were used. The outcomes were considered to be statistically significant when *P* < 0.05.

To clearly illustrate the relationships between metabolites, pre-diabetes and T2DM, the data provided in the publications were reprocessed. We calculated estimated means and SDs for each metabolite for which median and interquartile ranges were reported in the publications [18, 19]. Because the published data were presented in different forms, using means ± SD/SE or OR value, the outcome indicators were unified to better express the results. The mean ± SD of each metabolite provided in included studies was calculated as standardized mean difference (SMD), and then the SMD was converted to OR value using formula 1 [20, 21].

1 $$ \ln \kern0.5em OR= SMD\kern0.5em \ast \frac{\pi }{\sqrt{3}} $$

The mean and SD for ORs were obtained using SPSS 20.0 (IBM, Inc., Armonk, NY, USA) and converted outliers were removed when their values were larger than the mean plus five times SD [22]. The ORs were used to construct scatter diagrams with Graphpad Prism 7.0 (GraphPad Software, Inc., San Diego, USA), ensuring that there were at least three sets of data for each metabolite.

## Results

### Study selection

**Fig. 1:**
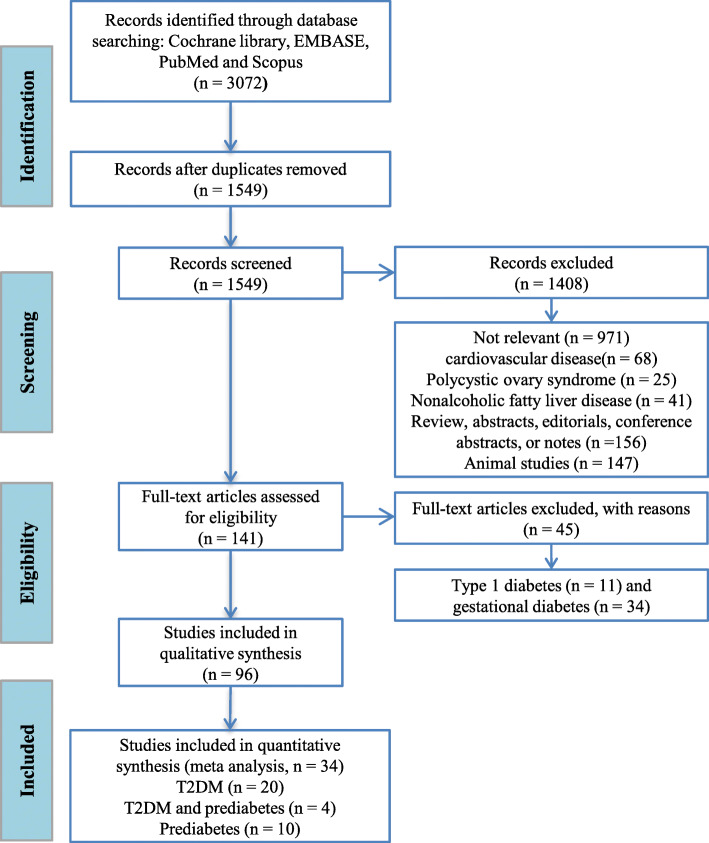
PRISMA flow diagram for the included studies of type 2 diabetes mellitus and pre-diabetes. After the removal of duplicates from the same database, 112 articles were found within the Cochrane Library, 984 within EMBASE, 689 within PubMed and 1287 within Scopus

A total of 3072 publications were identified from the database, and 1549 relevant articles remained after the removal of duplicate studies. A further 1408 publications were excluded after evaluating their titles and abstracts. These comprised 971 studies unrelated to the research topics; 68 that were on inflammation or cardiovascular diseases; 25 on polycystic ovary syndrome; 41 on non-alcoholic fatty liver disease; 156 were reviews, abstracts, editorials, conference papers or notes and 147 were performed on animals. Thus, 141 publications remained for assessment of the full text. After excluding studies of T1DM or GDM and qualitative research, 34 studies remained for inclusion in the meta-analysis, 20 of which were of T2DM, 10 were of pre-diabetes and 4 were of both T2DM and pre-diabetes. The PRISMA flow diagram for the meta-analysis is presented in Fig. 1.

### Quality assessment

The scores of for the studies included in this meta-analysis, generated using the NOS criteria, were shown in Table S1 and Table S2. The maximum score, awarded on the basis of eight risk assessment items [16], was nine stars. Studies with a score of five stars or more were regarded as of medium-to-high quality; otherwise, they were to be categorized as poor-quality and excluded. However, the lowest score was six stars. This implies that all the included studies were of medium-to-high quality, meaning that the data extracted were suitable for inclusion in the meta-analysis.

### Characteristics of the included studies

The characteristics of the included studies are shown in Table 1. They comprised 24 independent studies reporting data from 14131 healthy participants and 3499 T2DM patients [10, 11, 22–43]. All these studies compared T2DM patients with healthy participants. Four of the studies were prospective [26, 33, 37, 41] and four literatures were cohort studies [30, 31, 35, 43]. There were two cross-sectional studies [22, 40] and four follow-up studies [32, 34, 36, 42], and the rest were case-control studies. The results of most of the studies were presented as mean ± SD/SE, but some were presented as ORs.

**Table 1:** Characteristics of the data included in the meta-analysis of type 2 diabetes mellitus

Author-year-Journal	Study design	Population	Cases N	Control N	Biological sample	Detection method	Analytical method	Covariates	Metabolites
Zhu et al., 2011, Talanta [10]	Case-control study	Chinese	30	30	plasma	NPLC-TOF/MS	PCA, PLS-DA and ANOVA	Sex	(↑) LPC (16:0), LPC (18:2), LPC (18:0)
Liu et al., 2016, Sci Rep [11]	Case-control study	Chinese	15	15	plasma	1H NMR spectroscopy	PCA, PLS-DA, HCA and ROC curve analysis	Gender, age, BMI, SBP, DBP, total cholesterol, BUN and serum creatinine	(↑) Glucose, glycine (↓) Valine, leucine, alanine, isoleucine, arginine, proline, glutamine, threonine, tyrosine, creatine
Wang-sattler et al., 2017, Mol Syst Biol [22]	KORA S4 cross-sectional study	Augsburg and the surrounding towns and villages	KORA S4: 91 EPIC: 133 Prospective: 91	KORA S4: 866 EPIC: 1253 Prospective: 91	serum	LC-FIA-MS	Multivariate logistic regression and linear regression	Age, sex, BMI, PA, alcohol intake, smoking, SBP and HDL	(↓) Glycine, LPC (18:2)
Ha et al., 2012, Clin Endocrinol [23]	Case-control study	South Korean (men)	26	27	serum	UPLC/Q-TOF-MS	PLS-DA and ROC curve analysis	Age and BMI	(↑) Myristic acid (14:0) (−) Palmitic acid (16:0), stearic acid (18:0), arachidonic acid (20:4 ω6)
Menni et al., 2013, Diabetes [24]	Case-control study	British	115	1897	plasma	NA	Random intercept logistic regressions analysis	Age, BMI, batch effect and family relatedness	(↑) Valine, leucine, isoleucine, proline, mannose, glucose,2-hydroxybutyrate, lactate (↓) Citrulline, myristate (14:0),1,5-anhydroglucitol
Kujala et al., 2016, Front Med [25]	Case-control study	Older Finnish men	126	214	serum	NMR platform	Mann-Whitney U-test or Kruskal-Wallis test	Age	(↑) Valine, isoleucine, leucine
Peddinti et al., 2017, Diabetologia [26]	Botnia Prospective Study	Finland	146	397	serum	UHPLC-MS/MS and GC-MS	Multivariate logistic regression analysis	Age, sex, BMI, fasting insulin level and family history	(↑) Valine, isoleucine, glutamate, mannose, glucose (↓) Histidine, glutamine
Okekunle et al., 2017, Diabetes Res Clin Pract [27]	Case-control study	Chinese (Harbin)	50	50	serum	UPLC-TQ-MS	ANOVA and covariance analysis	Age, sex, BMI and insulin resistance	(↑) Glutamic acid, ornithine (↓) Serine, asparagine, glycine, threonine (−) Valine, leucine, isoleucine, tyrosine, phenylalanine, lysine, alanine, histidine, proline, citrulline, arginine, ethionine, glutamine
Abu Bakar et al., 2017, Mol Biosyst [28]	Nested-case control study	Malaysian	37	32	plasma	LC-MS and HPLC	The Kruskal-Wallis test, PLS-DA and ROC curve analysis	Age, gender, BMI and SBP	(↑) Valine, leucine, tyrosine, soleucine, phenylalanine, glutamine, glutamate, lysine, proline, palmitic acid, arachidonic acid, myristic acid (↓) Alanine, stearic acid
Andersson-Hall et al., 2018, J Diabetes Res [29]	6-years follow-up study	Women in Gothenburg	44	139	serum	NMR Spectroscopy	ANOVA and ANCOVA	BMI	(↑) Valine, leucine, tyrosine, isoleucine, glucose, mannose, acetoacetate, glycerol, 3-hydroxy-isobutyrate (↓) Glycine (−) Phenylalanine
Lee et al., 2016, Metabolomics [30]	KARE cohort (prospective) study	South Korean	517	924	serum	LC/MS/MS and LC-FIA-MS	Multivariable logistic regression and linear regression	Age, sex, BMI and HDL	(↓) Glycine, LPC a (18:2)
Floegel et al., 2013, Diabetes [31]	EPIC-Potsdam case-cohort study	The area of Potsdam in eastern Germany	800	2282	serum	FIA-MS/MS	Cox proportional hazards regression and PCA	Age, sex, alcohol intake, smoking, education, coffee intake, BMI and waist circumference	(↑) Phenylalanine (↓) Glycine, LPC a (18:2)
Gogna et al., 2015, Mol Biosyst [32]	Case-control study	South Indian Asians	165	128	serum	COSY, HSQC, HMQC, CPMG NMR spectra	PCA and PLS-DA	Age, sex and BMI	(↑) Valine, leucine, isoleucine, lysine, glutamine, phenylalanine, proline, threonine, histidine, glucose, lactate, glycerol
Lu et al., 2016, Diabetologia [33]	Prospective cohort study	Chinese	197	197	serum	LC-MS and GC-MS	OPLS-DA, ROC and conditional logistic regression analysis	Age, sex, BMI, smoking, status and history of hypertension	(↑) Valine, leucine, glycine, isoleucine, threonine, palmitic acid (16:0), stearic acid (18:0) (↓) Ornithine, proline, serine, glycerol
Palmer et al., 2015, J Clin Endocrinol Metab [34]	5-year follow-up study	European American, Hispanic and African American	76	70	plasma	MS/MS	Logistic regression analysis	Age, sex, ethnicity, recruitment site and BMI	(↑) Valine, leucine, isoleucine, tyrosine, phenylalanine, glutamine and glutamate (↓) Glycine, alanine (−) Serine, proline, histidine, methionine, ornithine, citrulline, arginine
Ng et al., 2012, Diabetologia [35]	Singapore Diabetes Cohort Study (SDCS)	Singaporean	44	46	urine	GC/MS and LC/MS	OPLS-DA, PCA and LASSO	Multiple hypotheses testing by controlling for FDR	(↑) L-serine, creatinine
Liu et al., 2017, Metabolomics [36]	14-years follow-up study	Southwest of the Netherlands	137	1434	plasma	LC-MS, NMR-COMP and NMR-LIPO	LASSO and ROC curve analysis	Age, sex, family history and BMI	(↑) Isoleucine, methionine, tyrosine, 1,5-anhydroglucitol, 2-hydroxybutyrate, lactate, glycerol
Merino et al., 2018, Diabetologia [37]	Prospective study	Framingham	95	1055	plasma	LC-MS/MS	LASSO and ROC curve analysis	Age, sex, BMI, fasting glucose and triacylglycerols	(↑) Phenylalanine (↓) Glycine
Li et al., 2017, Mol Biosyst [38]	Case-control study	Chinese	25	20	urine	GC-TOF-MS	PCA, OPLS-DA and ROC curve analysis	Age	(↓) Glycine (−) Serine, stearic acid, palmitic acid, 1,5-anhydroglucitol
Chou et al., 2018, J Chromatogr B [39]	Case-control study	Chinese (Harbin)	47	48	serum	GC-MS	ANOVA, ROC, PCA and PLS-DA analysis	Age, sex, smoking and alcohol consumption	(↑) Lactate (−) α-hydroxybutyrate
Wolak-Dinsmore et al., 2018, Clin Biochem [40]	Cross-sectional study	Groningen cohort-white	67	56	serum	LC-MS/MS and NMR spectroscopy	Multivariable linear regression analyses	Age and sex	(↑) Valine, leucine, isoleucine
Lu et al., 2019, Metabolites [41]	Prospective study	Chinese	144	144	serum	LC-MS	t-test, chi-square test and logistic regression	BMI, history of hypertension, smoking status, HDL-cholesterol and triglycerides	(↑) Valine, leucine, Isoleucine, phenylalanine, lysine, methionine, alanine (−) Threonine, histidine, glutamine, glycine, tyrosine, serine, proline
Lu et al., 2018, J Clin Endocrinol Metab [42]	Follow-up study	Singapore Chinese	144	144	serum	HPLC-QQQ-MS/GC	ROC and conditional logistic regression analysis	BMI, history of hypertension, smoking, physical activity, fasting status, triglycerides and HDL cholesterol	(↑) Myristic acid (14:0), palmitic acid (16:0), stearic acid (18:0), arachidonic acid (20:4n-6)
Friedrich et al., 2015, Metabolomics [43]	Longitudinal cohort study	North-east area of Germany	Men (87), Women (50)	Men (1266), Women (1306)	urine	NMR spectroscopy	Logistic regression and ROC curve analysis	Age and waist circumference	Women: (↑) alanine, glycine, glucose, lactate (↓) Creatinine Men: (↑) valine, glucose, lactate (−) Glycine

(↑), positive association; (↓), negative association; (−), no significant changes

*Abbreviations*: *GC* gas chromatography, *MS* mass spectrometry, *LC* liquid chromatography, *NMR* nuclear magnetic resonance spectroscopy, *UPLC-TQ-MS* ultra-high performance liquid chromatography tandem quadruple mass spectrometry, *FIA-MS/MS* flow injection analysis tandem mass spectrometry, *FIA-ESI-MS/MS* flow injection electrospray ionization tandem mass spectrometry, *UHPLC-MS/MS* high performance liquid chromatography tandem mass spectrometry, *KORA* Cooperative Health Research in the Region of Augsburg, *UPLC-QTOF-MS* ultra-high-performance liquid chromatography-quadrupole time-of-flight mass spectrometry, *LC-FIA-MS* liquid chromatography-flow injection analysis-mass spectrometry, *COSY* correlation spectroscopy, *CPMG* Car-Purcell-Meiboom-Gill, *HSQC and HMQC* heteronuclear and homonuclear single quantum coherence spectroscopy, *NMR-COMP* small molecular compounds window based NMR spectroscopy, *GC-TOF-MS* gas chromatography-time-of-flight mass spectrometry, *NPLC-TOF/MS* normal phase liquid chromatography coupled with time of flight mass spectrometry, *HPLC-QQQ-MS/GC* HPLC coupled triple quadrupole mass spectrometry, *GC-LC-FIA-MS/MS* gas chromatography-liquid chromatography-flow injection analysis mass spectrometry/mass spectrometry, *LASSO* least absolute shrinkage and selection operator, *OPLS-DA* orthogonal partial least squares-discriminant analysis, *ROC* receiver operating characteristic, *PCA* principal component analysis, *ANOVA* analysis of variance, *ANCOVA* analysis of covariance, *HCA* hierarchical cluster analysis, *FDR* false discovery rate, *BMI* body mass index, *SBP* systolic blood pressure, *DBP* diastolic blood pressure, *BUN* blood urea nitrogen, *HDL* high density lipoprotein, *LPC* lysophosphatidylcholine, *a* acyl

As shown in Table 2, there were 14 studies of pre-diabetes included in the meta-analysis, which contained a total of 2139 prediabetic patients and 4844 healthy controls [22, 24, 25, 29, 44–53]. Among these studies, one was a cross-sectional study [22], two were follow-up studies [29, 39], one was a longitudinal study [53], one was a cohort study [50], and the remaining nine were case-control studies. The participants in one study conducted in Gothenburg were only female [29]. The results of two of the studies were presented as medians and interquartile ranges [51, 52]. Therefore, the mean and SD of the metabolite concentrations mathematically were estimated [18, 19].

**Table 2:** Characteristics of the publications included in the meta-analysis of biomarkers in pre-diabetes

Author-year-Journal	Study design	Population	Cases N	Control N	Biological sample	Detection method	Analytical method	Covariates	Metabolites
Wang-sattler et al., 2017, Mol Syst Biol [22]	Cross-sectional study	Augsburg and the surrounding towns and villages	340	866	serum	LC-FIA-MS	Multivariate logistic regression and linear regression	Age, sex, BMI, PA, alcohol intake, smoking, SBP and HDL	(↓) Glycine
Menni et al., 2013, Diabetes [24]	Case-control study	British	192	1897	plasma	NA	Random intercept logistic regressions analysis	Age, BMI, batch effect and family relatedness	(↑) Valine, leucine, isoleucine (↓) Citrulline (−) proline, myristate (14:0)
Kujala et al., 2016, Front Med [25]	Case-control study	Older Finnish men	252	214	serum	NMR platform	Mann-Whitney U-test or Kruskal-Wallis test	Age	(↑) Valine (−) Leucine, isoleucine
Andersson-Hall et al., 2018, J Diabetes Res [29]	6-years follow-up study	Women in Gothenburg	46	139	serum	NMR Spectroscopy	ANOVA and ANCOVA	BMI and age	(↑) Valine, leucine, phenylalanine (↓) Glycine (−) Isoleucine, tyrosine
Cobb et al., 2016, Diabetes Care [44]	3-year follow-up study	RISC: 19 centers in 13 countries in Europe; DMVhi: Irish	RISC: 332; DMVhi: 183	RISC: 623; DMVhi; 485	plasma	LC-MS/MS	ROC and multiple logistic regression analyses	Age, sex, and BMI	(↑) Isoleucine, leucine, valine, phenylalanine, tyrosine, 2-aminoadipic acid (↓) Glycine (−) Serine
Tulipani et al., 2016, Clin Chim Acta [45]	Case-control study	Spanish (Málaga)	12	19	serum	LC-MS/MS, FIA-ESI-MS/MS	the R environment, Cytoscape	Age, sex, and BMI	(↑) Glutamate (↓) Glycine
Zeng et al., 2010, Metabolomics [46]	Case-control study	Chinese (Changsha)	34	24	plasma	GC/MS	PCA and PLS-DA	Age	(↑) Alanine (−) Leucine, proline, isoleucine, phenylalanine, tyrosine, serine
Butte et al., 2015, Am J Clin Nutr [47]	Case-control study	Hispanic America children	450	353	plasma	UPLC-MS and GC-MS	PCA and mixed-effects linear regression	Sex, age and Tanner stage	(↑) Alanine, glutamate, valine, isoleucine, leucine, tyrosine, phenylalanine, tryptophan, ornithine, carnitine (C0), propionylcarnitine (C3) (↓) Asparagine, glycine, serine, citrulline
Gao et al., 2016, Nutr Metab [48]	Case-control study	Canadian	30	20	serum	XEVO TQ MS system coupled with the Biocrates AbsoluteIDQ p180 kit	PLS-DA and ANOVA	BMI, age, total dietary calorie intake, and physical activity level	(↑) Leucine, isoleucine, valine, alpha-aminoadipic acid, propionylcarnitine (C3)
Kim et al., 2010, J Proteome Res [49]	Case-control study	South Korean	30	30	serum	UPLC-QTOF-MS	PCA and PLS-DA	Age	(↑) L-Valine, L-tyrosine, L-leucine, L-tryptophan, propionylcarnitine (C3), hexanoyl carnitine (C6) (↓) L-Carnitine (−) Myristate (14:0), palmitic (C16:0)
Lee et al., 2015, Obes Res Clin Pract [50]	Cohort study	South Korean	64	45	plasma	LC-MS/MS and FIA-MS/MS	ANOVA, logistic regression and ROC curve analyses	Age, BMI and waist circumference	(↑) Alanine, isoleucine, leucine, valine, phenylalanine, tyrosine, glutamate, proline, 2-amino adipic acid, carnitine (C0), propionylcarnitine (C3) (↓) Glycine, serine, asparagine, citrulline
Mastrangelo et al., 2016, Int J Obes [51]	Case-control study	Spanish	50	50	serum	LC-MS, GC-MS and capillary electrophoresis-mass spectrometry	OPLS-DA, PCA, univariate and multivariate analyses	BMI, age, fasting glucose and multiple testing	(↑) Valine, isoleucine, leucine, phenylalanine, tryptophan, tyrosine, alanine, proline, C03-carnitine
Newgard et al., 2009, Cell Metab [52]	Case-control study	African Americans	74	67	serum	MS and MS/MS	PCA	Age, race and sex	(↑) Valine, Aspartate/Asparagine, leucine/isoleucine, alanine, glutamate/glutamine, tyrosine, phenylalanine, proline, palmitic (C16:0), propionylcarnitine (C3), myristate (14:0) (↓) Glycine, citrulline (−) Serine
Ni et al., 2015, EBioMedicine [53]	Longitudinal study	Chinese (Shanghai)	50	12	serum	UPLC-QTOF-MS	OPLS-DA, ROC and logistic regression analysis, hierarchical clustering	Age, sex, BMI, HOMA-IR, and fasting glucose	(−) Myristate (14:0), palmitic C16:0

(↑), positive association; (↓), negative association; (−), no significant changes

*Abbreviations*: *RISC* Relationship Between Insulin Sensitivity and Cardiovascular Disease, *DMVhi* Diabetes Mellitus and Vascular Health Initiative, *LC-MS/MS* liquid chromatography coupled to tandem mass spectrometry, *FIA-ESI-MS/MS* flow injection electrospray ionization tandem mass spectrometry, *NMR* nuclear magnetic resonance spectroscopy, *GC/MS* gas chromatography/mass spectrometry, *LC-FIA-MS* liquid chromatography and flow injection analysis-mass spectrometry, *UPLC-QTOF-MS* ultra-performance liquid chromatograph coupled to quadruple time-of-flight mass spectrometry, *ROC* receiver operating characteristic, *ANOVA* analysis of variance, *ANCOVA* analysis of covariance, *PCA* principal component analysis, *PLS-DA* partial least squares-discriminant analysis, *OPLS-DA* orthogonal partial least squares-discriminant analysis, *BMI* body mass index, *HOMA-IR* homeostasis model assessment-insulin resistance, *LPC* lysophosphatidylcholine, *a* acyl

### Metabolites analyses

#### Characteristics of the metabolites studied

**Fig. 2:**
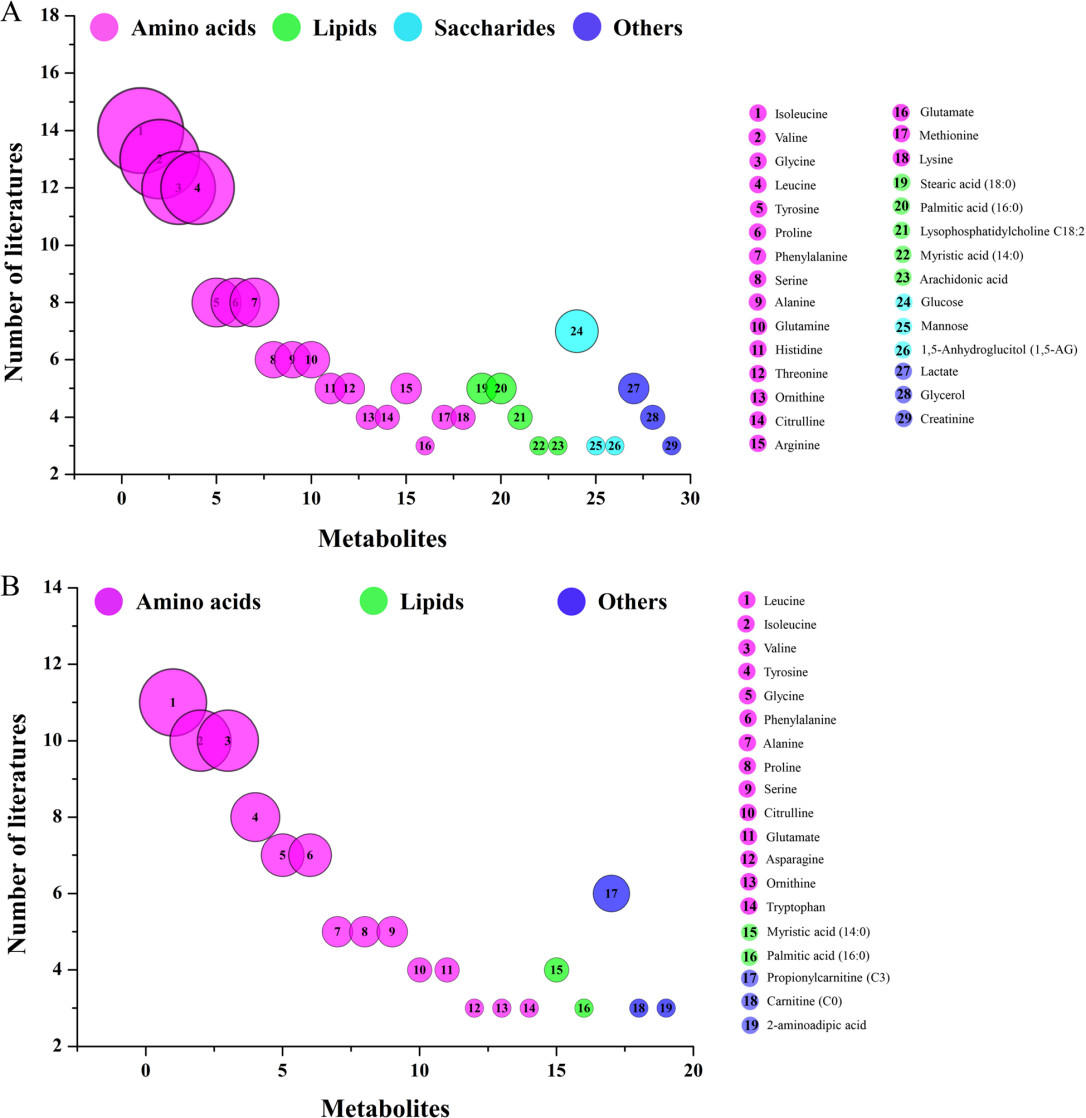
Bubble diagram of the publications on type 2 diabetes mellitus (**a**) and pre-diabetes (**b**)

Metabolites including amino acids, lipids, saccharides and others were analyzed in the 24 studies of T2DM. The frequencies of analysis of each metabolite in the 24 studies were counted and metabolites quantified in three or more studies are shown as a bubble diagram (Fig. 2a). The four categories of metabolite are shown in pink, green, blue and purple, respectively. The ordinal numbers on the bubbles represent different metabolites and the size of each bubble is indicative of the number of studies in which it was analyzed. Eighteen amino acids, five lipids, three saccharides and three other metabolites were assayed. Thus, the most studied metabolites were amino acids, of which the four most commonly analyzed were isoleucine, valine, glycine and leucine, in 14, 13, 12 and 12 studies, respectively. Metabolites studied on less than three articles were excluded, as summarized in Table S3.

For pre-diabetes, the number of metabolites studied in publications was significantly lower in the 14 studies included than for T2DM, as shown in Fig. 2b. There were 14 amino acids, 2 lipids and 3 other metabolites. The top three most commonly analyzed amino acids were leucine, isoleucine and valine, which were studied on 11, 10 and 8 occasions, respectively. Metabolites studied on less than three articles were excluded, as summarized in Table S4.

#### Analysis of metabolites associated with T2DM

On the basis of data extracted with means ± SD/SE forest plots for each metabolite were created using Review Manager 5.3. Because the dimensions and units used in the studies differed, SMDs were used for the forest plot outputs. For the T2DM studies, because the *I*^2^ values for glycine and tyrosine were 29 and 43%, respectively, with a *P* value in the *Q* test > 0.1, fixed effect models were used to calculate combined effect sizes. Moreover, the *I*^2^ values for valine, leucine, isoleucine, proline, glutamate, lysine, phenylalanine, alanine, histidine and serine were > 90% (Table S5). That is, random effect models were used for these metabolites [17, 54].

**Fig. 3:**
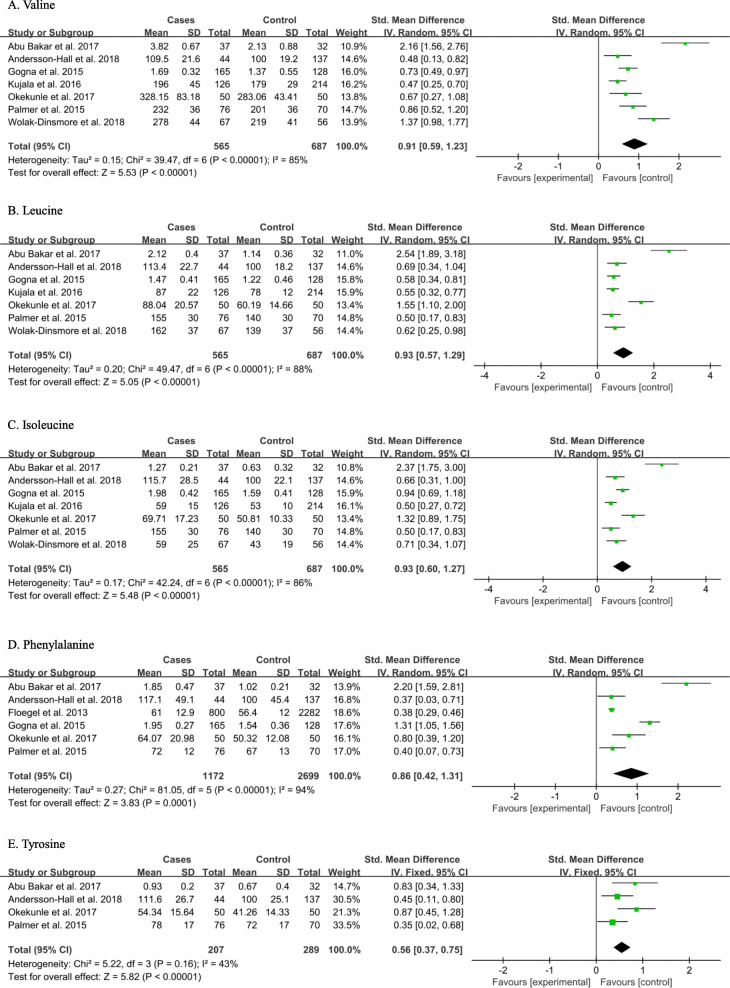
Pooled analysis of valine (**a**), leucine (**b**), isoleucine (**c**), phenylalanine (**d**) and tyrosine (**e**) in the serum or plasma of type 2 diabetes mellitus patients and control participants

As shown in Fig. 3, the concentrations of BCAAs and aromatic amino acids (AAAs) were significantly higher in the serum and plasma of T2DM patients than in control participants. The SMDs of valine (0.91 [0.59, 1.23], *P* < 0.00001), leucine (0.93 [0.57, 1.29], *P* < 0.00001), isoleucine (0.93 [0.60, 1.27], *P* < 0.00001), phenylalanine (0.86 [0.42, 1.31], *P* = 0.0001) and tyrosine (0.56 [0.37, 0.75], *P* < 0.00001) were statistically significant. Additionally, the concentration of glycine (− 0.42 [− 0.49, − 0.34], *P* < 0.00001) was lower and those of proline (0.50 [0.18, 0.82], *P* = 0.002), glutamate (0.63 [0.19, 1.07], *P* = 0.005) and lysine (0.84 [0.28, 1.40], *P* = 0.003) were higher, in the serum and plasma of patients with T2DM than in control participants (Fig. S1). Thus, valine, leucine, isoleucine, tyrosine, glycine, proline, glutamate and lysine could be considered as biomarkers of T2DM according to their forest plots and the first five of these are likely to be most useful, given the associated *P* values.

#### Analysis of metabolites associated with pre-diabetes

For the prediabetic studies, the *I*^2^ values for isoleucine, proline, citrulline, 2-aminoadipic acid and lysine were less than 50%, with the *P* value for the *Q* test > 0.1 and therefore fixed effect models were used to calculate the combined effect sizes. The *I*^2^ values for glycine, alanine, glutamate, serine and palmitic acid (C16:0), leucine, valine, tyrosine, phenylalanine, propionylcarnitine (C3), carnitine (C0), asparagine, tryptophan and myristate (C14:0) were > 50% (Table S6). Therefore, random effect models were used.

**Fig. 4:**
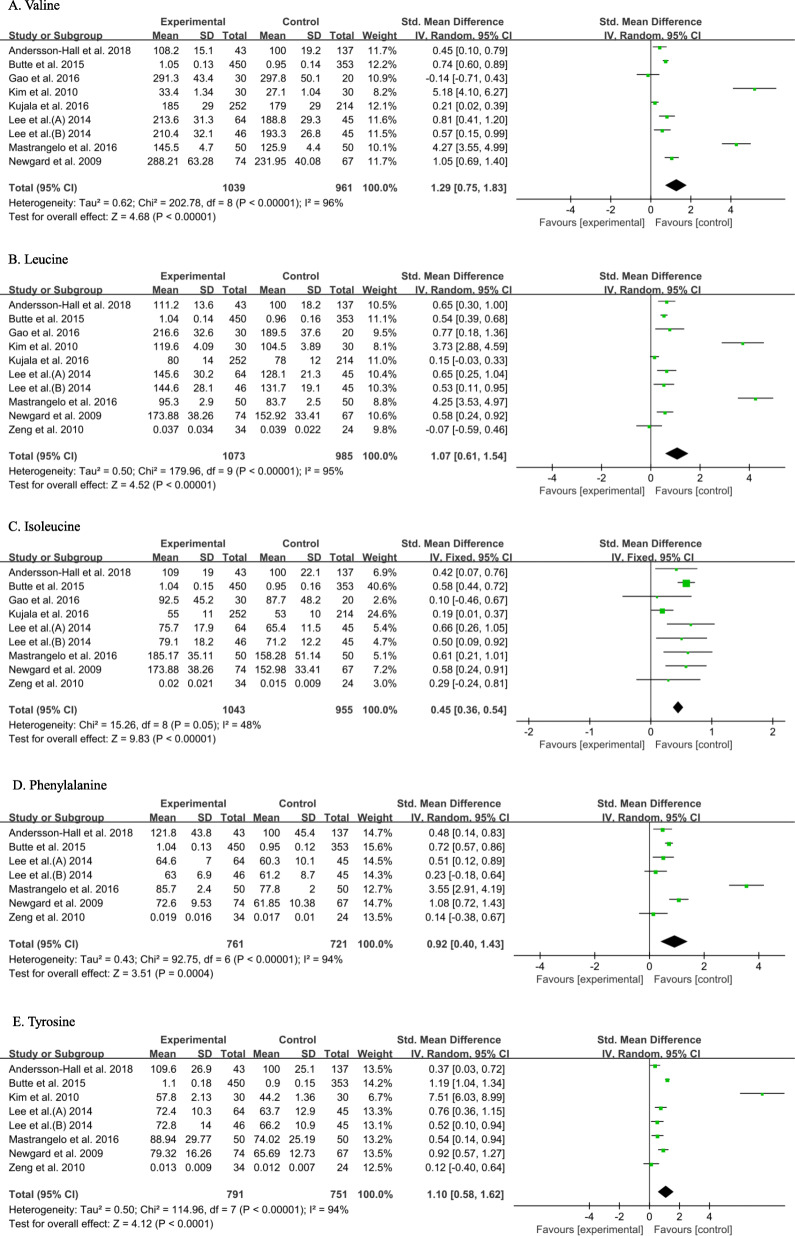
Pooled analysis of valine (**a**), leucine (**b**), isoleucine (**c**), phenylalanine (**d**) and tyrosine (**e**) in serum and plasma of pre-diabetes and control groups. Studies with several populations comparing patients with pre-diabetes and controls are described by the author name followed by A or B to indicate, for example, subdivision according to sex

As shown in Fig. 4 and Table S6, the concentrations of valine (1.29 [0.75, 1.83], *P* < 0.00001), leucine (1.07 [0.61, 1.54], *P* < 0.00001), isoleucine (0.45 [0.36, 0.54], *P* < 0.00001), phenylalanine (0.92 [0.40, 1.43], *P* = 0.0004) and tyrosine (1.10 [0.58, 1.62], *P* < 0.0001) were significantly higher in the serum and plasma of prediabetic patients than in control participants. The concentration of glycine (− 0.76 [− 1.00, − 0.51], *P* < 0.00001) was lower, while those of proline (0.41 [0.23, 0.59], *P* < 0.00001), glutamate (0.61 [0.20, 1.02], *P* = 0.004) and lysine (0.36 [0.24, 0.49], *P* < 0.00001) were higher in the serum or plasma of prediabetic patients than in control participants (Fig. S2). Furthermore, there were statistically significant differences in the concentrations of serine, citrulline, 2-aminoadipic acid and palmitic acid (C16:0) in the serum or plasma between prediabetic and healthy participants, as shown in Fig. S3 and Table S6. The concentrations of alanine (0.57 [0.30, 0.83], *P* < 0.0001), 2-aminoadipic acid (0.69 [0.43, 0.95], *P* < 0.00001), propionylcarnitine (C3) (1.65 [0.83, 2.48], *P* < 0.0001) and palmitic acid (C16:0) (0.85 [0.44, 1.26], *P* < 0.0001) in the serum or plasma of prediabetic patients were higher than those of healthy participants, while concentrations of serine (− 0.37 [− 0.70, − 0.04], *P* = 0.03) and citrulline (− 0.37 [− 0.49, − 0.25], *P* < 0.00001) were lower. This implies that isoleucine, glycine, proline, glutamate, lysine, serine, citrulline, 2-aminoadipic acid and palmitic acid (C16:0) may represent biomarkers of prediabetes.

### Integrative analysis of the metabolite biomarkers

Forest plots were only constructed for metabolites analyzed in at least three studies included in the meta-analysis, but these may not represent the most widely applicable assays. For example, IFG and IGT were only assessed in prediabetic patients in one study [44], but more than three datasets for each metabolite can be more reliably integrated to reflect the features of pre-diabetes. Therefore, we conducted integrative profiling to scientifically combine all the data provided in the included studies.

**Fig. 5:**
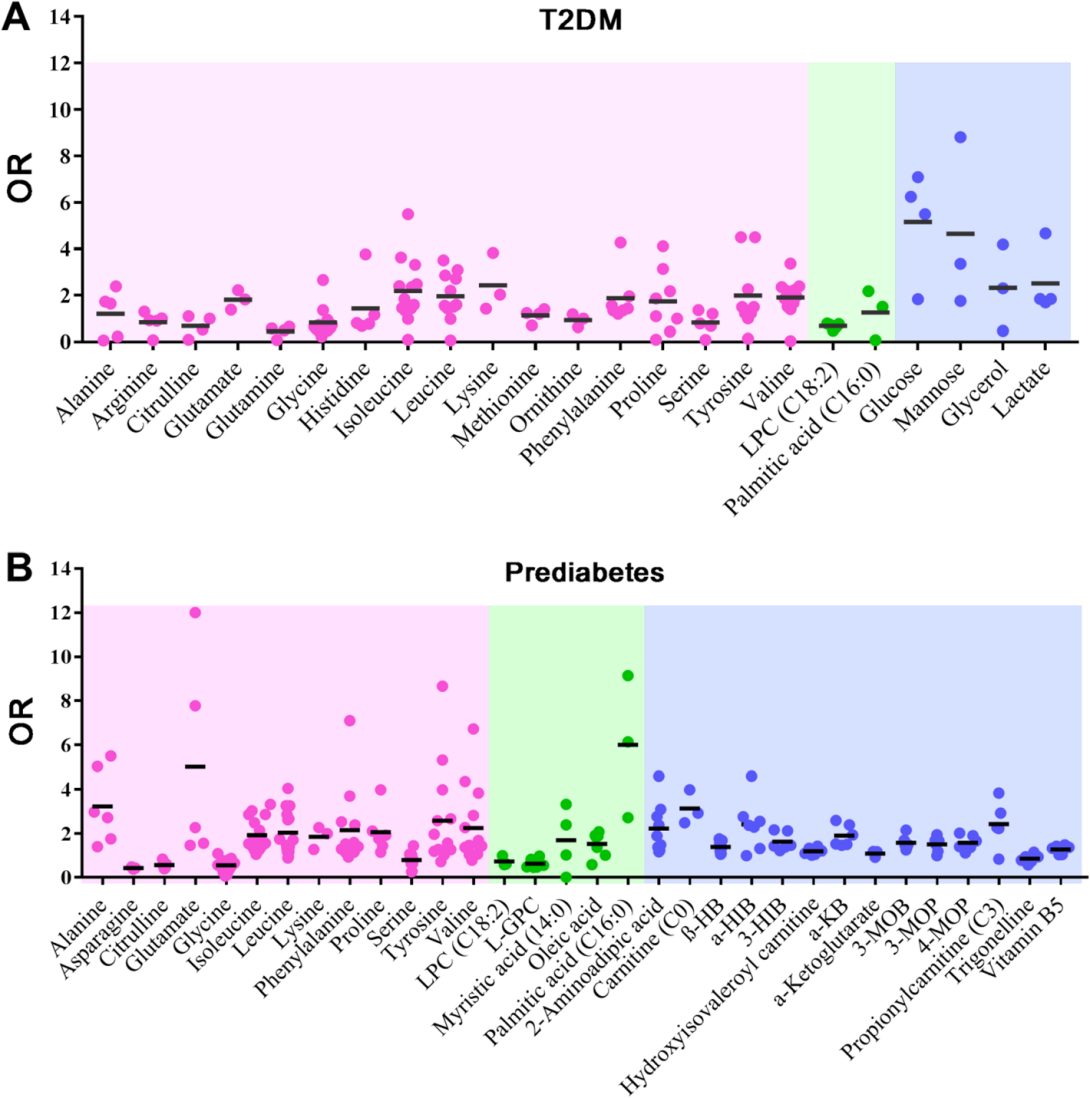
Metabolite profiling diagrams. **a** Metabolite profiling diagrams for metabolites in type 2 diabetes mellitus patients. **b** Metabolite profiling diagrams for metabolites in pre-diabetic patients. Abbreviations: OR, odds ratio; LPC, lysophosphatidylcholine; L-GPC, linoleoyl-glycerophospho-choline; α-HIB, α-hydroxyisobutyric acid; β-HB, β-hydroxybutyric acid; 4-MOP, 4-methyl-2-oxopentanoic acid; 3-MOP, 3-methyl-2-oxopentanoic acid; 3-MOB, 3-methyl-2-oxobutyric acid; α-KB, ketobutyric acid; β-HIB, β-hydroxyisobutyric acid

The ORs for each metabolite provided in the included publications were analyzed to reflect the characteristics of the disease biomarkers, excluding publications containing outliers. As shown in Fig. 5a, 23 metabolites of those analyzed in T2DM patients remained after those with outliers had been excluded. In the scatter diagram, the dots represent the ORs and the colors represent the types of metabolite. The mean ORs for the isoleucine (OR = 2.19), leucine (OR = 1.95), valine (OR = 1.91), phenylalanine (OR = 1.88), lysine (OR = 2.43), arginine (OR = 0.83), methionine (OR = 1.14), glycine (OR = 0.88), tyrosine (OR = 1.99), serine (OR = 0.83), proline (OR = 1.74), alanine (OR = 1.21), glutamate (OR = 1.81), citrulline (OR = 0.69), histidine (OR = 1.44), glutamine (OR = 0.46) and ornithine (OR = 0.94) were significant. The mean ORs for lysophosphatidylcholine (LPC C18:2) and palmitic acid (C16:0) were 0.68 and 1.26, respectively. The mean ORs for glucose and mannose were 5.17 and 4.65, respectively. And the mean ORs for lactate and glycerol were 2.51 and 2.32, respectively. The mean ORs of all the metabolites were demonstrated the characteristic metabolic profile of T2DM.

In the pre-diabetes studies, as shown in Fig. 5b, 32 metabolites were analyzed, comprising 13 amino acids, 5 lipids and 14 other metabolites. The mean ORs of isoleucine (OR = 1.92), leucine (OR = 2.03), valine (OR = 2.24), phenylalanine (OR = 2.15), lysine (OR = 1.84), asparagine (OR = 0.42), glycine (OR = 0.55), tyrosine (OR = 2.57), serine (OR = 0.79), proline (OR = 2.05), alanine (OR = 3.23), glutamate (OR = 5.01), citrulline (OR = 0.56), LPC (C18:2) (OR = 0.73), palmitic acid (C16:0) (OR = 5.99), oleic acid (OR = 1.52), linoleoyl-glycerophospho-choline (OR = 0.62), myristic acid (C14:0) (OR = 1.68), hydroxyisovaleroyl carnitine (OR = 1.18), propionylcarnitine (C3) (OR = 2.41), carnitine (C0) (OR = 3.12), 2-aminoadipic acid (OR = 2.21), α-hydroxyisobutyric acid (OR = 2.40), β-hydroxybutyric acid (OR = 1.38), 4-methyl-2-oxopentanoic acid (OR = 1.57), 3-methyl-2-oxopentanoic acid (OR = 1.49), 3-methyl-2-oxobutyric acid (OR = 1.57), ketobutyric acid (OR = 1.90), 3-hydroxyisobutyric acid (OR = 1.62), vitamin B5 (OR = 1.27), α-ketoglutarate (OR = 1.08) and trigonelline (OR = 0.85) were significant. Unlike T2DM, no saccharides were analyzed. The mean ORs for all the metabolites were constructed to indicate the characteristics of the metabolic profile for pre-diabetes.

From Fig. 5, obviously, alanine, citrulline, glutamate, glycine, isoleucine, leucine, lysine, phenylalanine, proline, serine, tyrosine and valine amino acids, LPC (C18:2) and palmitic acid (C16:0) were statistically similar between T2DM/pre-diabetes patients and healthy controls. The obvious difference in pre-diabetes and T2DM indicates that these disease stages are associated with distinct and quantified metabolic biomarker profiles. In particular, the metabolic biomarkers alanine, glutamate and palmitic acid (C16:0) were significantly different in pre-diabetes and T2DM, which suggests that quantified concentrations of this three metabolites are potential for use as integrative biomarkers for the differentiation of pre-diabetes and T2DM.

## Discussion

The use of a single biomarker to diagnose a disease lacks specificity because multiple disease processes are likely to affect its concentration. Additionally, the main disadvantage of the simple addition of other biomarkers is that their discriminative ability typically overlaps, also limiting the use of this approach [55]. Some studies involving diet were excluded, because the higher intake of metabolites might falsely raise their levels in metabolomics [56]. The use of single biomarkers is limited by the effects of external factors, such as diet. Furthermore, risk models containing biomarkers derived from the pathways directly affected by the disease itself may not demonstrate high predictive value. We believe that the integration of data regarding a number of biomarkers more accurately predict the occurrence of pre-diabetes/T2DM, and map the patient’s current state in a precise manner, which might prevent the further development of T2DM, diabetic macro- and microphaties.

The present meta-analysis, which included 34 independent studies reported data from 14,515 healthy participants, 3499 patients of T2DM and 2139 with pre-diabetes, was performed based on both original OR and OR converted from SMD value. SMD could reflect the original data of each study, and reduce the deviations caused by different methods in included studies. Therefore, the comparability and reliability of meta-analysis are acceptable [21]. There were 23 metabolites concerning T2DM and 32 metabolites concerning pre-diabetes based on included studies. From Fig. 5, obviously, 12 amino acids, LPC (C18:2) and palmitic acid (C16:0) were statistically similar between T2DM/pre-diabetes patients and healthy controls. Metabolite biomarkers of T2DM and pre-diabetes revealed that the levels of alanine, glutamate and palmitic acid (C16:0) are significantly different in T2DM and pre-diabetes. These findings could reflect the different status of pre-diabetes and T2DM, and could provide an important reference for clinical diagnosis and treatment of pre-diabetes and early T2DM, which might prevent the further development of T2DM and reduce the incidence of diabetes complications.

Integrated profiling reflects a set of biomarkers in the context of a network, instead of considering only single or isolated biomarkers. As shown in Fig. S4, the pathogenesis of T2DM is complex and involves many signaling pathways, which has not yet been fully elucidated. Integration hence of the pre-existing metabolite biomarkers may be useful for the prevention and diagnosis of T2DM and pre-diabetes. This method of analysis is suitable for the integration of a number of types of data; for instance, both amino acid biomarkers, belonging to centralized data with strong regularity and a wide range of metabolic biomarkers, belonging to dispersive and isolated data with irregularity. The goals of most studies is improving the diagnosis rate of pre-diabetes and early T2DM, which could reduce the incidence of T2DM and diabetes complications through early intervention treatment. Integrative profiling of metabolic biomarkers should be able to provide reliable references for the selection of biomarkers suitable for the prediction and diagnosis of T2DM and pre-diabetes in the future. It is more potential clinical valuable for high incidence of diabetes (such as China and India) to explore metabolite biomarkers profile for identification and diagnosis of pre-diabetes and T2DM [3]. For the abnormal amino acid and lipid profiles (low levels of metabolites such as glycine, serine and LPC (18:2)), is it possible to increase their levels through external intake to reduce the incidence of pre-diabetes or T2DM? It is worthwhile to design experiments to verify this conjecture at the animal level in future. In further research, conducting clinical, multi-center cohort or prospective observation trials are necessary and important research works.

Although it has shown that quantified metabolic biomarkers could reflect T2DM and pre-diabetes, there were some limitations to the approach used. First, some relevant studies may not have been retrieved from the databases using the search terms described. Second, there were fewer studies of some of the metabolites and there is likely to be a publication bias in favor of positive findings, which may have introduced bias into our analysis. Third, all the information regarding the samples and data collected were derived from the included studies, so the potential confounding factors present in these studies, such as ethnicity, region, education and physical health of the participants might have affected the study results. Although the accuracy of the meta-analysis results was affected by the original research data form included studies, the conclusions of this study were obtained from the meta-analysis conducted in strict compliance with the included criteria and the PRISMA guidelines.

## Conclusions

Quantified multiple metabolite biomarkers are useful strategy to differentiate pre-diabetes and T2DM, and we believe that it has potential clinical value for the diagnosis of T2DM.

## Supplementary Information

**Additional file 1.**

## Data Availability

The datasets used and/or analysed during the current study are available from the corresponding author on reasonable request.

## Abbreviations

T2DM: Type 2 diabetes mellitus
BCAAs: Branched chain amino acids
PRISMA: Preferred Reporting Items for Systematic Reviews and Meta-Analyses
GDM: Gestational diabetes mellitus
T1DM: Type 1 diabetes mellitus
IGT: Impaired glucose tolerance
IFG: Impaired fasting glucose
NOS: Newcastle-Ottawa Scale
SD: Standard deviation
SE: Standard error
OR: Odds ratio
CI: Confidence interval
SMD: Standardized mean difference
LPC: Lysophosphatidylcholine
