# Supplementary material for: Metabolite biomarkers of type 2 diabetes mellitus and pre-diabetes: a systematic review and meta-analysis
Source: BMC Endocr Disord. 2020 Nov 23;20:174. doi: 10.1186/s12902-020-00653-x (PMC7685632; doi:10.1186/s12902-020-00653-x)
Supplement: Supplementary file 1 — Additional file 1. [file 12902_2020_653_MOESM1_ESM.doc]

**Supplementary Materials**

**Metabolite biomarkers of type 2 diabetes mellitus and pre-diabetes: a systematic review and meta-analysis**

Jianglan Long^1,2^, Zhirui Yang^1^, Long Wang^3^, Yumei Han^4^, Cheng Peng^2^, Can Yan^5*^ and Dan Yan^1*^

**Author Affiliations**:

^1^ Beijing Key Laboratory and Joint Laboratory for International Cooperation of Bio-characteristic Profiling for Evaluation of Rational Drug Use, Capital Medical University Affiliated Beijing Shijitan Hospital, Beijing 100038, China

^2^ Chengdu University of Traditional Chinese Medicine, Chengdu 611130, China

^3^ Department of Applied Mathematics and Statistics, Johns Hopkins University, Baltimore, MD 21218, USA

^4^ Beijing Physical Examination Center, Beijing 100077, China

^5^ Guangzhou University of Chinese Medicine, Guangzhou 510006, China

***Corresponding author**:

Can Yan, Email: Yanc020@126.com; Dan Yan, Email: pharmsci@126.com

**Description of the mechanism diagram**

Abnormal concentrations of metabolites may affect the progression of T2DM by regulating the insulin signaling pathway [1]. Insulin resistance is associated with dysfunctional metabolism and abnormal concentrations of metabolites [2]. The results of the present meta-analysis show that the concentrations of BCAAs and AAAs are significantly higher in the serum or plasma of T2DM and prediabetic patients. Over recent years, BCAAs (especially leucine) have become a focus of research. Leucine deprivation results in increases in lipolysis and the expression of β-oxidation genes in white adipose tissue and thermogenesis in brown adipose tissue [3], which can be prevented by the cerebroventricular injection of leucine [4]. Furthermore, in an experimental study [5], the researchers found that rats consuming a high-fat diet containing BCAAs were significantly more insulin resistant than rats consuming normal feed or an high-fat diet alone.

As shown in Fig. S4, amino acids stimulate mTOR, inhibit AKT and further increase or decrease the activation or expression of downstream mediators. First, the transcription factor peroxisome proliferator activated receptor alpha (PPARα) is inhibited by multi-step inhibitory modification by mTOR complex 1 (mTORC1), which dysregulates lipid metabolism [6]. Additionally, the PPARγ coactivator-1α (PGC-1α), PPARγ, sterol regulatory element-binding protein (SREBP)-1 transcription factors and lipin 1 are activated by multi-step stimulatory modification by mTORC1, which promotes mitochondrial metabolism, adipogenesis, lipogenesis and lipid synthesis [7]. Second, lower expression of phosphatidylinositol-4-phosphate 5-kinases (PIP5K), 6-phosphofructo-2-kinase/fructose-2,6-biphosphatase (PFKFB-2) and hormone-sensitive lipase (HSL) may suppress glucose transport, glycolysis and lipolysis [8]. Deactivation of salt-inducible kinase 2 (SIK2), cyclic adenosine monophosphate (cAMP) response element binding protein (CREB) binding protein (CBP) [9] and forkhead box protein O1 (FoxO1) transcription factor [10] might increase gluconeogenesis. However, the higher expression of SREBP transcription factor may promote fatty acid synthesis [11]. Additionally, amino acids or glucose could downregulate liver kinase B1 (LKB1) by reducing the AMP/ATP ratio [12], which would inhibit the activity of AMP-activated protein kinase (AMPK) and its downstream substrates [13], having further effects on metabolism. For example, a reduction in glucose transporter protein 4 (GLUT4) expression could inhibit glucose transport [14], higher expression of SREBP-1 and CREB could promote lipogenesis and gluconeogenesis [15], an increase in acetyl-coenzyme A carboxylase (ACC) activity and a reduction in carnitine palmitoyltransferase 1 (CPT1) activity could result in the inhibition of fatty acid oxidation [16] and lower expression of PFKFB3 and adipose triglyceride lipase (ATGL) might inhibit glycolysis and lipolysis [17]. All the molecules can regulate glucose transport or glucose and lipid metabolism.

Additionally, cytokines and hormones can induce insulin resistance by regulating the insulin signaling pathway. These include tumor necrosis factor (TNF) [18], orosomucoid and inflammatory cytokines [19], intercellular adhesion molecule 1 (ICAM-1) and vascular cell adhesion molecule 1 (VCAM-1) [20]. Alterations in the concentrations of amino acids, lipids and glucose in T2DM patients may also affect signaling, but the mechanism of insulin resistance requires further research for its full elucidation.

Numerous environmental factors affect the progression of T2DM and may confound studies of this disease. For example, dietary composition, especially with regard to complex carbohydrates, has differing effects in T2DM patients and controls [21]. A high-fiber diet down-regulates blood glucose and lipid concentrations in T2DM patients [22]. Furthermore, disease progression has a substantial influence on the concentrations of metabolites in T2DM patients, as well as in pig and rat models [23,24]. However, the studies included in the present analysis did not record the stage of progression of the T2DM patients. Sex, age and other factors also have an impact on metabolite concentrations, because these variables affect metabolism [25]. Furthermore, both single-center and multi-center studies from multiple countries around the world (including China, Singapore and Germany) were included, which might have introduced a number of confounders affecting the results.

**References**

1. Woods YL, Petrie JR, Sutherland C: Dissecting insulin signaling pathways: individualised therapeutic targets for diagnosis and treatment of insulin resistant states. Endocr Metab Immune Disord Drug Targets 2009, 9(2):187-198.

2. Lynch CJ, Adams SH: Branched-chain amino acids in metabolic signalling and insulin resistance. Nat Rev Endocrinol 2014, 10(12):723-736.

3. Cheng Y, Meng Q, Wang C, Li H, Huang Z, Chen S, et al: Leucine deprivation decreases fat mass by stimulation of lipolysis in white adipose tissue and upregulation of uncoupling protein 1 (UCP1) in brown adipose tissue. Diabetes 2010, 59(1):17-25.

4. Banks WA, Kastin AJ: Leucine modulates peptide transport system-1 across the blood-brain barrier at the stereospecific site within the central nervous system. J Pharm Pharmacol 1991, 43(4):252-254.

5. Newgard CB, An J, Bain JR, Muehlbauer MJ, Stevens RD, Lien LF, et al: A branched-chain amino acid-related metabolic signature that differentiates obese and lean humans and contributes to insulin resistance. Cell Metab 2009, 9(4):311-326.

6. Djouadi F, Brandt JM, Weinheimer CJ, Leone TC, Gonzalez FJ, Kelly DP: The role of the peroxisome proliferator-activated receptor alpha (PPAR alpha) in the control of cardiac lipid metabolism. Prostaglandins Leukot Essent Fatty Acids 1999, 60(5-6):339-343.

7. Blanchard PG, Festuccia WT, Houde VP, St-Pierre P, Brule S, Turcotte V, et al: Major involvement of mTOR in the PPARgamma-induced stimulation of adipose tissue lipid uptake and fat accretion. J Lipid Res 2012, 53(6):1117-1125.

8. Houddane A, Bultot L, Novellasdemunt L, Johanns M, Gueuning MA, Vertommen D, et al: Role of Akt/PKB and PFKFB isoenzymes in the control of glycolysis, cell proliferation and protein synthesis in mitogen-stimulated thymocytes. Cell Signal 2017, 34:23-37.

9. Patel K, Foretz M, Marion A, Campbell DG, Gourlay R, Boudaba N, et al: The LKB1-salt-inducible kinase pathway functions as a key gluconeogenic suppressor in the liver. Nat Commun 2014, 5:4535.

10. Puigserver P, Rhee J, Donovan J, Walkey CJ, Yoon JC, Oriente F, et al: Insulin-regulated hepatic gluconeogenesis through FOXO1-PGC-1alpha interaction. Nature 2003, 423(6939):550-555.

11. Horton JD, Goldstein JL, Brown MS: SREBPs: activators of the complete program of cholesterol and fatty acid synthesis in the liver. J Clin Invest 2002, 109(9):1125-1131.

12. Leclerc I, Rutter GA: AMP-activated protein kinase: a new beta-cell glucose sensor?: Regulation by amino acids and calcium ions. Diabetes 2004, 53 Suppl 3:S67-74.

13. Jakobsen SN, Hardie DG, Morrice N, Tornqvist HE: 5'-AMP-activated protein kinase phosphorylates IRS-1 on Ser-789 in mouse C2C12 myotubes in response to 5-aminoimidazole-4-carboxamide riboside. J Biol Chem 2001, 276(50):46912-46916.

14. Calera MR, Martinez C, Liu H, Jack AK, Birnbaum MJ, Pilch PF: Insulin increases the association of Akt-2 with Glut4-containing vesicles. J Biol Chem 1998, 273(13):7201-7204.

15. Li Y, Xu S, Mihaylova MM, Zheng B, Hou X, Jiang B, et al: AMPK phosphorylates and inhibits SREBP activity to attenuate hepatic steatosis and atherosclerosis in diet-induced insulin-resistant mice. Cell Metab 2011, 13(4):376-388.

16. Park SH, Gammon SR, Knippers JD, Paulsen SR, Rubink DS, Winder WW: Phosphorylation-activity relationships of AMPK and acetyl-CoA carboxylase in muscle. J Appl Physiol 2002, 92(6):2475-2482.

17. Domenech E, Maestre C, Esteban-Martinez L, Partida D, Pascual R, Fernandez-Miranda G, et al: AMPK and PFKFB3 mediate glycolysis and survival in response to mitophagy during mitotic arrest. Nat Cell Biol 2015, 17(10):1304-1316.

18. Hotamisligil GS, Peraldi P, Budavari A, Ellis R, White MF, Spiegelman BM: IRS-1-mediated inhibition of insulin receptor tyrosine kinase activity in TNF-alpha- and obesity-induced insulin resistance. Science 1996, 271(5249):665-668.

19. Schmidt MI, Duncan BB, Sharrett AR, Lindberg G, Savage PJ, Offenbacher S, et al: Markers of inflammation and prediction of diabetes mellitus in adults (Atherosclerosis Risk in Communities study): a cohort study. Lancet 1999, 353(9165):1649-1652.

20. Meigs JB, Hu FB, Rifai N, Manson JE: Biomarkers of endothelial dysfunction and risk of type 2 diabetes mellitus. Jama 2004, 291(16):1978-1986.

21. Joshi SR, Bhansali A, Bajaj S, Banzal SS, Dharmalingam M, Gupta S, et al: Results from a dietary survey in an Indian T2DM population: a STARCH study. BMJ Open 2014, 4(10):e005138.

22. R.T I, Kolawole B, E.O O, Salawu A, Ajose O, S A, et al: A controlled comparison of the effect of a high fiber diet on the glycaemic and lipid profile of Nigerian clinic patients with type 2 diabetes. Pakistan J Nutr 2007, 6(2):111-116

23. Jensen-Waern M, Andersson M, Kruse R, Nilsson B, Larsson R, Korsgren O, et al: Effects of streptozotocin-induced diabetes in domestic pigs with focus on the amino acid metabolism. Lab Anim 2009, 43(3):249-254.

24. Piccolo BD, Graham JL, Stanhope KL, Fiehn O, Havel PJ, Adams SH: Plasma amino acid and metabolite signatures tracking diabetes progression in the UCD-T2DM rat model. Am J Physiol Endocrinol Metab 2016, 310(11):E958-969.

25. Froberg K, Pedersen PK: Sex differences in endurance capacity and metabolic response to prolonged, heavy exercise. Eur J Appl Physiol Occup Physiol 1984, 52(4):446-450.


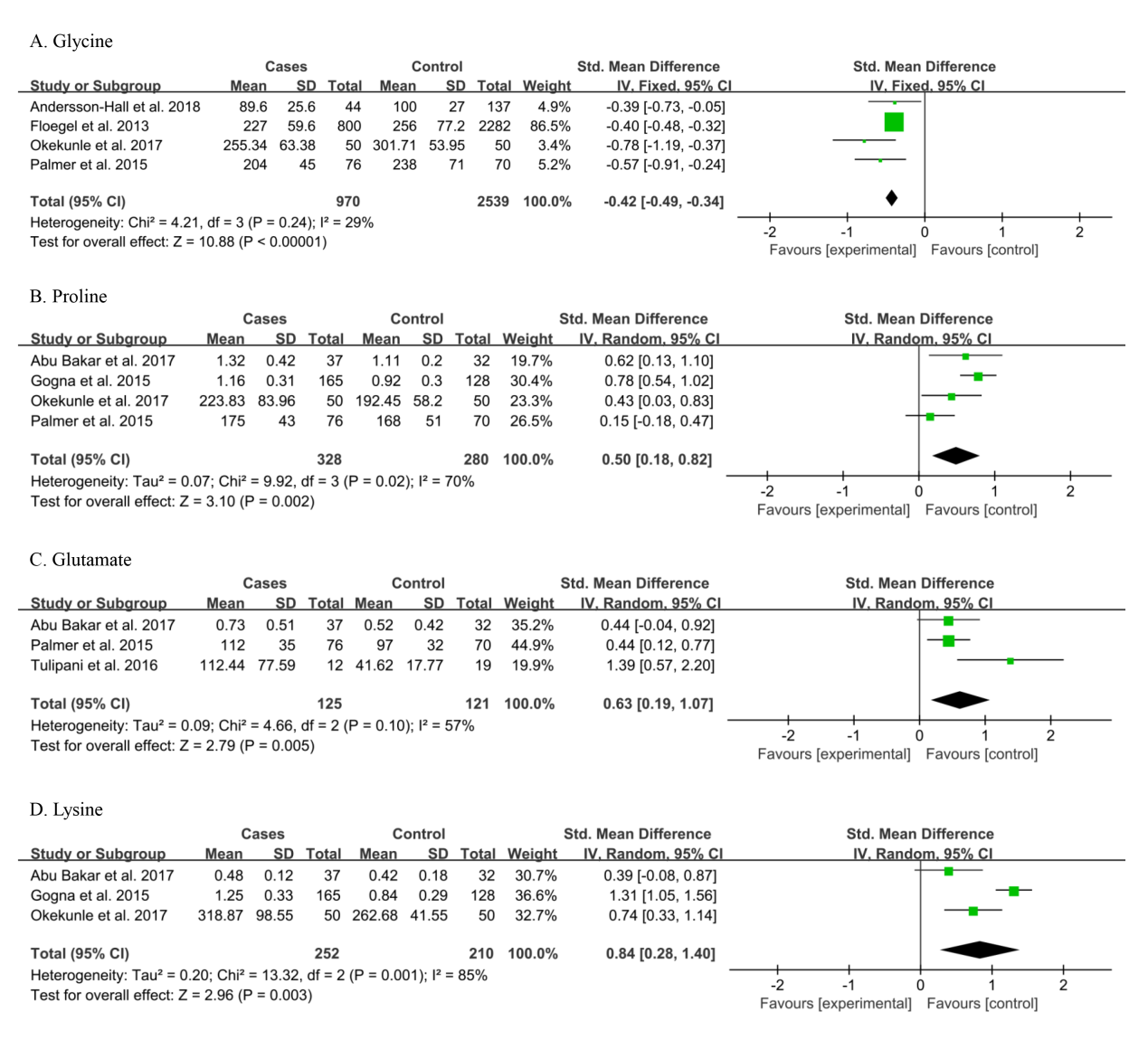


Fig. S1: Pooled analysis of glycine (A), proline (B), glutamate (C) and lysine (D) in serum or plasma samples from patients with type 2 diabetes mellitus and control participants.


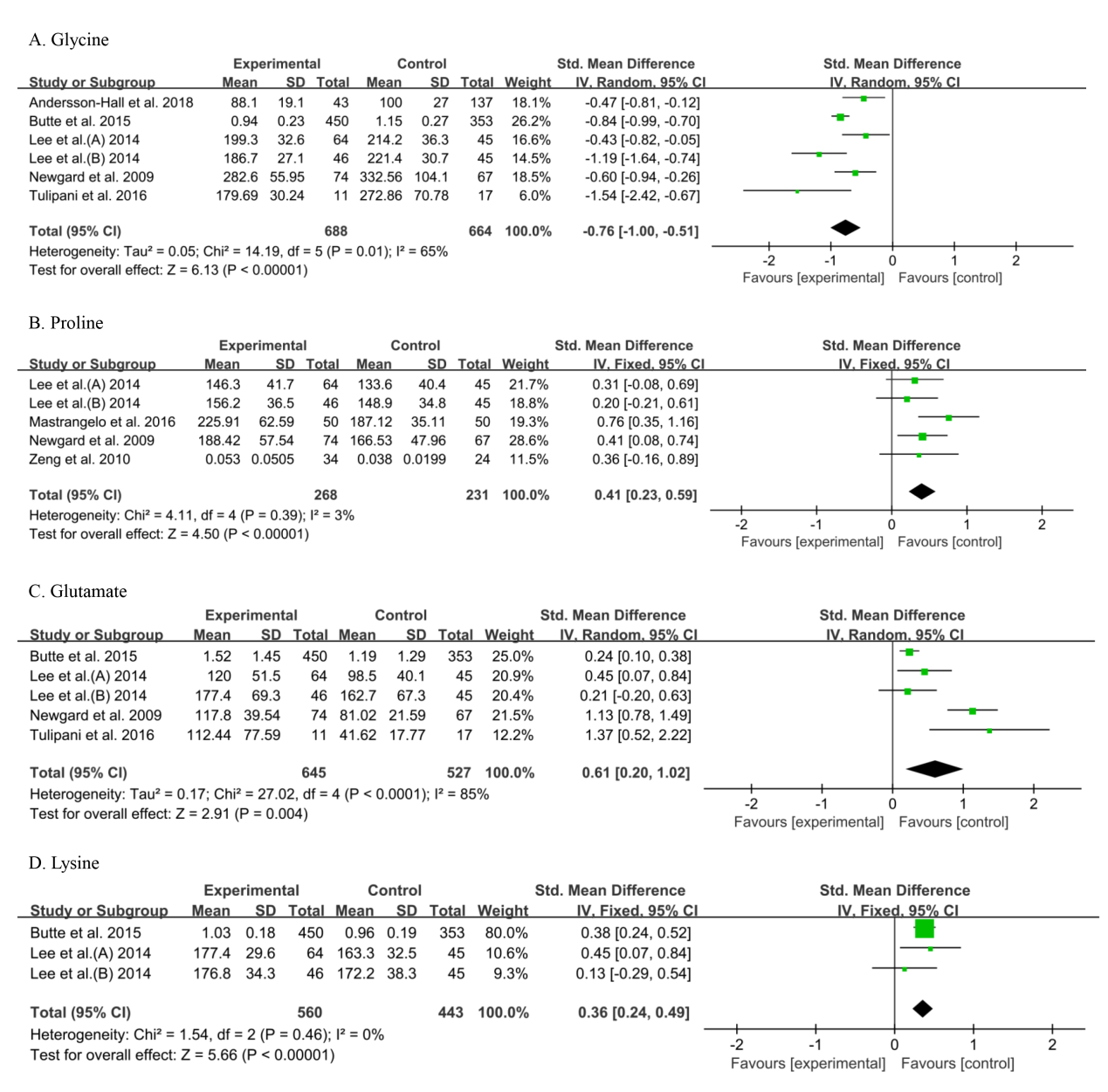


Fig. S2: Pooled analysis of glycine (A), proline (B), glutamate (C) and lysine (D) in serum or plasma samples from prediabetic patients and control participants.

Studies of several populations that compared patients with prediabetes and controls are labeled using the author’s name, followed by A or B to indicate subdivision according to the duration of follow-up.


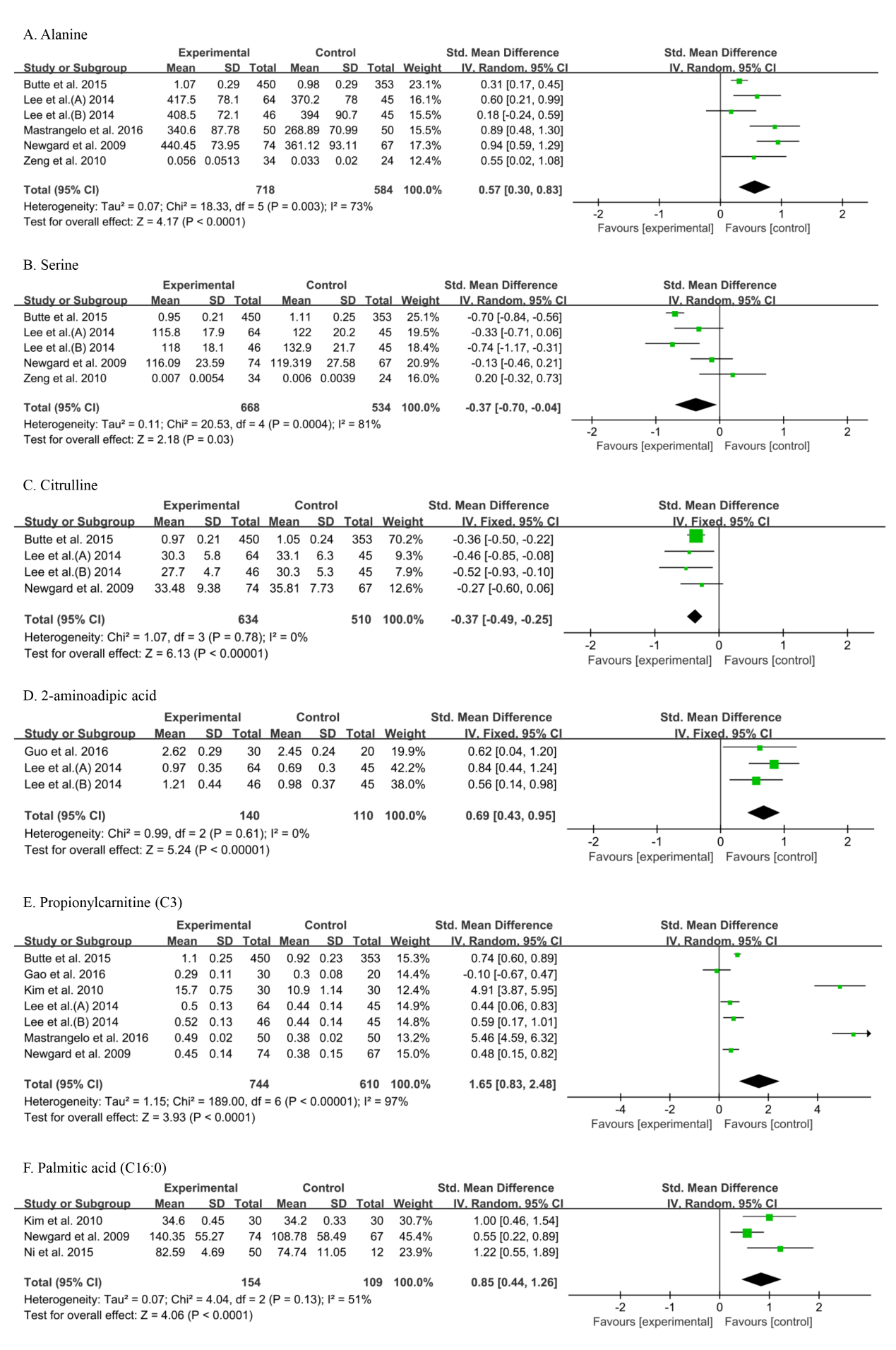


Fig. S3: Pooled analysis of alanine (A), serine (B), citrulline (C), 2-aminoadipic acid (D), propionylcarnitine (C3) (E) and palmitic acid (C16:0) (F) in serum or plasma samples from prediabetic patients and control participants.

Studies of several populations that compared patients with diabetes and controls are labeled using the author’s name, followed by A or B to indicate subdivision according to sex.


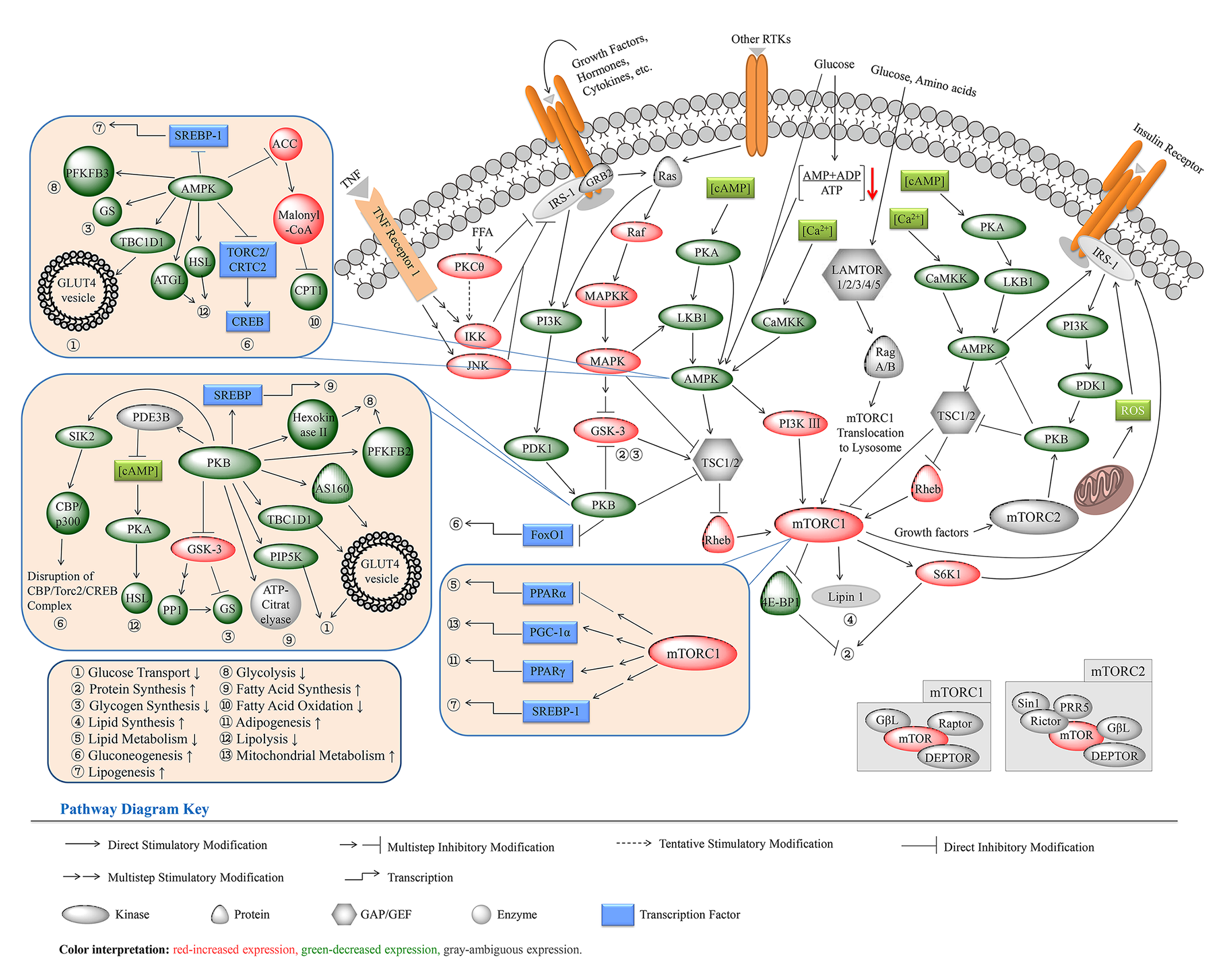


Fig. S4. Signaling pathways shown to be dysregulated in type 2 diabetes mellitus. Abbreviations: GRB2, growth factor receptor-bound protein 2; IRS-1, insulin receptor substrate 1; PI3K, phosphatidylinositol 3-kinase; PI3K Ⅲ, class 3 phosphatidylinositol 3-kinase; PDK1, phosphoinositide-dependent kinase 1; PKB, protein kinase B; GSK-3, glycogen synthesis kinase 3; Ras, Ras protein; MAPK, mitogen-activated protein kinase; MAPKK, mitogen-activated protein kinase; Raf, Raf kinase; PKA, protein kinase A; LKB1, liver kinase B1; ADP, adenosine diphosphate; ATP, adenosine triphosphate; AMP, adenosine monophosphate; AMPK, AMP-activated protein kinase; TSC1/2, tuberous sclerosis complex proteins 1 and 2; Rheb, Rheb protein; CaMKK, calmodulin-dependent protein kinase; mTORC1/2, mammalian target of rapamycin complex 1/2; S6K1, ribosomal protein S6 kinase 1; 4E-BP1, initiation factor 4E binding protein; Rag A/B, Rag protein A/B; LAMTOR1/2/3/4/5, late endosomal/lysosomal adaptor, MAPK and mTOR activator 1/2/3/4/5; TNF, tumor necrosis factor; RTK, receptor tyrosine kinase; GS, glutamine synthetase; PP1, phosphatase 1; HSL, hormone-sensitive lipase; PIP5K, phosphatidylinositol-4-phosphate 5-kinases; TBC1D1, Tre-2/Bub2/Cdc16 domain family, member 1; AS160, Rab GTPase-activating protein; GLUT4, glucose transporter protein 4; PFKFB, 6-phosphofructo-2-kinase/fructose-2,6-biphosphatase; PDE3B, phosphodiesterase type 3B; SIK2, salt-inducible kinase 2; ATGL, adipose triglyceride lipase; CREB, cAMP-response element binding protein; CBP, CREB binding protein; CRTC2, CREB regulated transcription coactivator 2; FOXO1, forkhead box protein O1; SREBP, sterol regulatory element-binding protein; PPAR, peroxisome proliferator-activated receptor; PGC-1α, PPAR gamma coactivator 1α; ACC, acetyl coenzyme A carboxylase; CPT1, carnitine palmitoyltransferase 1; IKK, inhibitor of nuclear factor kappa-B kinase; JNK, c-Jun N-terminal kinase.

**Table S1.** Risk of bias assessment for case control studies of included studies, conducted using the Newcastle-Ottawa Scale.

| **Studies** | | **Selection** | | | | **Comparability** | **Exposure** | | |  |
| --- | --- | --- | --- | --- | --- | --- | --- | --- | --- | --- |
| **First author** | **Year** | **Is the case definition adequate** | **Representativeness of the cases** | **Selection of controls** | **Definition of controls** | **Comparability of cases and controls on the basis of the design or analysis** | **Ascertainment of exposure** | **Same method of ascertainment for cases and controls** | **Non-**  **response rate** | **Total**  **score** |
| Wolak-Dinsmore J | 2018 | ★ | ★ |  | ★ | ★★ | ★ | ★ | ★ | 8 |
| Okekunle AP | 2017 | ★ |  |  | ★ | ★★ | ★ | ★ | ★ | 7 |
| Abu Bakar MH | 2017 | ★ | ★ |  | ★ | ★★ | ★ | ★ | ★ | 8 |
| Menni C | 2013 | ★ | ★ | ★ | ★ | ★★ |  | ★ | ★ | 8 |
| Kujala UM | 2016 | ★ | ★ |  | ★ | ★★ | ★ | ★ | ★ | 8 |
| Liu X | 2016 | ★ |  |  | ★ | ★★ | ★ | ★ | ★ | 7 |
| Floegel A | 2013 | ★ | ★ | ★ | ★ | ★★ | ★ | ★ | ★ | 9 |
| Gogna N | 2015 | ★ | ★ |  | ★ | ★★ | ★ | ★ | ★ | 8 |
| Li L | 2017 | ★ |  |  | ★ | ★★ | ★ | ★ | ★ | 6 |
| Wang-sattler R | 2012 | ★ | ★ |  | ★ | ★★ | ★ | ★ | ★ | 8 |
| Zhu C | 2011 | ★ |  |  | ★ | ★★ | ★ | ★ | ★ | 6 |
| Ha CY | 2012 | ★ |  |  | ★ | ★★ | ★ | ★ | ★ | 7 |
| Cobb J | 2016 | ★ | ★ | ★ | ★ | ★★ | ★ | ★ | ★ | 9 |
| Tulipani S | 2016 | ★ | ★ |  | ★ | ★★ | ★ | ★ | ★ | 8 |
| Zeng M | 2010 | ★ | ★ |  | ★ | ★★ |  | ★ | ★ | 7 |
| Newgard CB | 2009 | ★ | ★ | ★ | ★ | ★★ | ★ | ★ | ★ | 9 |
| Ni Y | 2015 | ★ |  |  | ★ | ★★ | ★ | ★ | ★ | 7 |
| Butte NF | 2015 | ★ | ★ |  | ★ | ★★ | ★ | ★ | ★ | 8 |
| Mastrangelo A | 2016 | ★ | ★ |  | ★ | ★★ | ★ | ★ | ★ | 8 |
| Kim JY | 2010 | ★ | ★ |  | ★ | ★★ | ★ | ★ | ★ | 8 |
| Lee A | 2015 | ★ | ★ |  | ★ | ★★ | ★ | ★ | ★ | 8 |
| Gao X | 2016 | ★ |  |  | ★ | ★★ | ★ | ★ | ★ | 7 |
| Chou J | 2018 | ★ |  |  | ★ | ★★ | ★ | ★ | ★ | 8 |

Note: 9 is the maximum score. A higher score corresponds to a lower risk of bias and a score of < 5 indicates a high risk of bias.

**Table S2.** Risk of bias assessment for cohort studies of included studies, conducted using the Newcastle-Ottawa Scale.

| **Studies** | | | **Selection** | | | | **Comparability** | **Outcome** | | |  |
| --- | --- | --- | --- | --- | --- | --- | --- | --- | --- | --- | --- |
| **First author** | | **Year** | **Representativeness of the exposed cohort** | **Selection of the un exposed cohort** | **Ascertainment of exposure** | **Demonstration that outcome of interest was not present at start of study** | **Comparability of cohorts on the basis of the design or analysis** | **Assessment of outcome** | **Was follow-up long enough for outcomes to occur** | **Adequacy of follow up of cohorts** | **Total**  **score** |
| Ng DP | | 2012 | ★ | ★ | ★ | ★ | ★★ | ★ | ★ | ★ | 9 |
| Peddinti G | | 2017 | ★ | ★ | ★ | ★ | ★★ | ★ | ★ | ★ | 9 |
| Lee HS | 2016 | | ★ | ★ | ★ | ★ | ★★ |  | ★ | ★ | 8 |
| Lu Y | 2016 | | ★ | ★ |  | ★ | ★★ | ★ | ★ | ★ | 8 |
| Palmer ND | 2015 | | ★ | ★ | ★ | ★ | ★★ |  | ★ | ★ | 8 |
| Liu J | 2017 | | ★ | ★ | ★ | ★ | ★★ | ★ | ★ | ★ | 9 |
| Merino J | 2018 | | ★ | ★ | ★ | ★ | ★★ | ★ | ★ | ★ | 9 |
| Lu Y | 2018 | | ★ | ★ |  | ★ | ★★ | ★ | ★ | ★ | 8 |
| Friedrich N | 2015 | | ★ | ★ | ★ | ★ | ★★ | ★ | ★ | ★ | 9 |
| Andersson-Hall U | 2018 | | ★ | ★ | ★ | ★ | ★★ | ★ | ★ | ★ | 8 |
| Lu YH | 2019 | | ★ | ★ |  | ★ | ★★ | ★ | ★ | ★ | 8 |

Note: 9 is the maximum score. A higher score corresponds to a lower risk of bias and a score of < 5 indicates a high risk of bias.

**Table S3.** Characteristics of the metabolites studied in fewer than three publications on type 2 diabetes mellitus.

| **Author-year-Journal** | **Study design** | **Population** | **Cases N** | **Control N** | **Biological sample** | **Detection method** | **Analytical method** | **Covariates** | **Metabolites** |
| --- | --- | --- | --- | --- | --- | --- | --- | --- | --- |
| Ng et al., 2012, Diabetologia | Singapore Diabetes Cohort Study (SDCS) | Singaporean | 44 | 46 | urine | GC/MS and LC/MS | OPLS-DA, PCA and LASSO | Multiple hypotheses testing by controlling for FDR | Dodecanoic acid (C12:0), xylitol, octanol, 3,5-dimethoxymandelic amide, N-acetylglutamine, benzamide, phosphoric acid, 2-hydroxyadipic acid, ribonic acid, hydroxyphenylacetic acid, sarcosine, salicyluric acid, uric acid, ß-hydroxybutyric acid, cis-aconitic acid, 2-ketogluconic acid, threitol, 3-hydroxyhippuric acid, succinic acid, D-glucuronic acid, pseudouridine |
| Okekunle et al., 2017, Diabetes Res Clin Pract | Case-control study | Chinese (Harbin) | 50 | 50 | serum | UPLC-TQ-MS | ANOVA and covariance analysis | Age, sex, BMI and insulin resistance | Glutamic acid, tryptophan, asparagine, taurine |
| Abu Bakar et al., 2017, Mol Biosyst | Nested-case control study | Malaysian | 37 | 32 | plasma | LC-MS and HPLC | The Kruskal-Wallis test, PLS-DA and ROC curve analysis | Age, gender, BMI and SBP | Aminobutyric acid, hydroxyisobutyric acid, pyroglutamic acid, oleic acid, linoleic acid, SM (d18:1/16:0), SM (d18:2/16:0), acetylcarnitine, propionylcarnitine, succinylcarnitine, decatrienoylcarnitine, dodecenoylcarnitine, lauroylcarnitine, tetradecenoylcarnitine, myristoylcarnitine, hexadecadienoylcarnitine, palmitoleoylcarnitine, linoleylcarnitine, oleylcarnitine, stearoylcarnitine |
| Menni et al., 2013, Diabetes | Case-control study | British | 115 | 1897 | plasma | NA | Random intercept logistic regressions analysis | Age, BMI, batch effect and family relatedness | N-acetylglycine, dimethylarginine, malate, 3-methyl-2-oxobutyrate, adrenate (22:4n6), 3-methyl-2-oxovalerate, myristoleate (14:1n5), 4-methyl-2-oxopentanoate, 15-methylpalmitate, 10-Heptadecenoate (17:1n7), arachidonate (20:4n6), myristate (14:0), , palmitoleate (16:1n7), heptanoate (7:0), pentadecanoate (15:0), 5-dodecenoate (12:1n7), pelargonate (9:0), palmitoyl sphingomyelin, cholesterol, fructose, arabinose, octanoylcarnitine |
| Peddinti et al., 2017, Diabetologia | Botnia Prospective Study | Finland | 146 | 397 | serum | UHPLC-MS/MS and GC-MS | Multivariate logistic regression analysis | Age, sex, BMI, fasting insulin level and family history | Trehalose, bilirubin (E,E), α-Tocopherol, X-16071 (RI: 3616, M: 146.2), α-HB (Q), X-13537 (RI: 5292, M: 295.3), X-13452 (RI: 3606, M: 192.2), X-12844 (RI: 4168, M: 539.3), X-12802 (RI: 2731, M: 318.2) |
| Liu et al., 2016, Sci Rep | Case-control study | Chinese | 15 | 15 | plasma | 1H NMR spectroscopy | PCA, PLS-DA, HCA and ROC curve analysis | Gender, age, BMI, SBP, DBP, total cholesterol, BUN and serum creatinine | Isopropanol, acetate, creatine, 3-methylhistidine |
| Lee et al., 2016, Metabolomics | KARE cohort (prospective) study | South Korea | 517 | 924 | serum | LC/MS/MS and LC-FIA-MS | Multivariable logistic regression and linear regression | Age, sex, BMI and HDL | PC ae 36:0, hexadecanoylcarnitine (C16) |
| Floegel et al., 2013, Diabetes | EPIC-Potsdam case-cohort study | German | 800 | 2282 | serum | FIA-MS/MS | Cox proportional hazards regression and PCA | Age, sex, alcohol intake, smoking, education, coffee intake, BMI and waist circumference | Hexose, PC ae 34:3, PC ae 40:6, PC ae 42:5, PC ae 44:4, PC ae 44:5, PC aa 32:1, PC aa 36:1, PC aa 38:3, PC aa 40:5, SM (16:1) |
| Gogna et al., 2015, Mol Biosyst | Case-control study | South Indian Asians | 165 | 128 | serum | COSY, HSQC, HMQC, CPMG NMR spectra | PCA and PLS-DA | Age, sex and BMI | Saturated fatty acids, 3-hydroxybutyric acid, choline, 3,7-dimethyluric acid, pantothenic acid, myoinositol, sorbitol |
| Lu et al., 2016, Diabetologia | Prospective cohort study | Chinese | 197 | 197 | serum | LC-MS and GC-MS | OPLS-DA, ROC and conditional logistic regression analysis | Age, sex, BMI, smoking, status and history of hypertension | Amino acids: 2-aminooctanoic acid, aminomalonic acid, hippuric acid  Lipids: oleic acid (18:1), linoleic acid (18:2)LPE (20:3), LPE (20:5), LPG (12:0), LPI (16:1), LPI (18:1), LPI (18:2), LPI (20:3), LPI (20:4), LPI (22:6)  Carbohydrates: CDP-glucose, D-galactose, gluconate  Others: 9-decenoylcarnitine (C10), lactic acid,pyruvate, urea, 1,3-propanediol |
| Palmer et al., 2015, J Clin Endocrinol Metab | 5-year follow-up study | European American, Hispanic and African American | 76 | 70 | plasma | MS/MS | Logistic regression analysis | Age, sex, ethnicity, recruitment site and BMI | Asparagine and aspartate |
| Liu et al., 2017, Metabolomics | 14-years follow-up study | Southwest of the Netherlands | 137 | 1434 | plasma | LC-MS, NMR-COMP and NMR-LIPO | LASSO and ROC curve analysis | Age, sex, family history and BMI | Lipids: PC (O-34:2), TG (48:0), TG (48:1), TG (50:5)  Others: 2-oxoglutaric acid, glycine betaine, pyruvate |
| Merino et al., 2018, Diabetologia | Prospective study | Framingham | 95 | 1055 | plasma | LC-MS/MS | LASSO and ROC curve analysis | Age, sex, BMI, fasting glucose and triacylglycerols | Taurine |
| Li et al., 2017, Mol Biosyst | Case-control study | Chinese | 25 | 20 | urine | GC-TOF-MS | PCA, OPLS-DA and ROC curve analysis | Age | Tagatose, ethanolamine, succinic acid, aconitic acid, isocitric acid, 4-hydroxybutyrate, glycolic acid, threonic acid, hydroxylamine, hippuric acid, 3-hexenedioic acid, 2-deoxyerythritol |
| Wang-sattler et al., 2017, Mol Syst Biol | KORA S4 cross-sectional study | Augsburg and the surrounding towns and villages | 91 | 866 | serum | LC-FIA-MS | Multivariate logistic regression and linear regression | Age, sex, BMI, PA, alcohol intake, smoking, SBP and HDL | Acetylcarnitine C2 |
| Zhu et al., 2011, Talanta | Case-control study | Chinese | 30 | 30 | plasma | NPLC-TOF/MS | PCA, PLS-DA and ANOVA | Sex | Lipids: LPC (18:1), LPC (20:4), LPC (16:0/18:2), LPC (16:0/18:0), LPC (18:0/20:4), PE (16:0/18:1), PE (16:0/20:4), PE (pC18:0/20:4), PG (18:0/18:2), PI (16:0/18:0), PI (18:0/20:4), PI (18:0/22:6), PS (18:0/18:0), SM (18:1/16:0), SM (18:0/20:2) |
| Lu et al., 2018, J Clin Endocrinol Metab | Follow-up study | Singapore Chinese | 144 | 144 | serum | HPLC-QQQ-MS/GC | ROC and conditional logistic regression analysis | BMI, history of hypertension, smoking, physical activity, fasting status, triglycerides and HDL cholesterol | Lipids: LPI (16:0), LPI (16:1), LPI (18:0), LPI (18:2), LPI (20:4), LPI (22:6), palmitoleic acid (16:1n-7), oleic acid (18:1n-9), linoleic acid (18:2n-6), α-linolenic acid (18:3n-3), γ-linolenic acid (18:3n-6), gondoic acid (20:1n-11), eicosadienoic acid (20:2n-6), nitine, dihomo-g-linolenic acid (20:3n-6), mead acid (20:3n-9), eicosapentaenoic acid (20:5n-3), adrenic acid (22:4n-6), clupanodonic acid (22:5n-3), osbond acid (22:5n-6), docosahexaenoic acid (22:6n-3)  Others: myo-Inositol, C20:5-carnitine, 3-hydroxybutyrylcarnitine, L-carnitine, dodecanoylcarnitine, octanoylcar decanoylcarnitine, tetradecanoylcarnitine, 3-hydroxydodecanoylcarnitne, 3-hydroxytetradecanoylcarnitine, hexadecanoylcarnitine, 3-hydroxyhexadecanoylcarnitine, 3-hydroxy-9-hexadecenoylcarnitine, |
| Friedrich et al., 2015, Metabolomics | Longitudinal cohort study | North-east area of Germany | Men (87), Women (50) | Men (1266), Women (1306) | urine | NMR spectroscopy | Logistic regression and ROC curve analysis | Age and waist circumference | Women: tau-methylhistidine, acetate, carnitine, ethanol, formate, glycolate, N,N-dimethylglycine, trimethylamine N-oxide, trigonelline, urea, 3-hydroxyisovalerate  Men: 4-hydroxyphenylacetate |
| Ha et al., 2012, Clin Endocrinol (Oxf) | Case-control study | South Korean (men) | 26 | 27 | serum | UPLC/Q-TOF-MS | PLS-DA and ROC curve analysis | Age and BMI | Lipids: dodecanoic acid (12:0), palmitoleic acid (c16:1), oleic acid (c18:1 ω9), oleic acid (c18:1 ω7), linoleic acid (c18:2 ω6), γ-linolenic acid (c18:3 ω6), eicosadienoic acid (c20:2 ω6), dihomo-c-linolenic acid (c20:3 x6), α-linolenic acid (c18:3 ω3), eicosapentaenoic acid (c20:5 ω3), docosahexaenoic acid (c22:6 ω3), δ-9 desaturase (18:1 ω9/18:0), δ-9 desaturase (16:1 ω7/16:0),δ-6 desaturase (18:3 ω6/18:2 ω6), δ-5 desaturase (20:4 ω6/20:3 ω6) |
| Andersson-Hall et al., 2018, J Diabetes Res | 6-years follow-up study | Women in Gothenburg | 44 | 139 | serum | NMR Spectroscopy | ANOVA and ANCOVA | BMI | Citrate, pyruvate, acetoacetate, 3-hydroxy-isobutyrate |
| Chou et al., 2018, J Chromatogr B Analyt Technol Biomed Life Sci | Case-control study | Chinese (Harbin) | 47 | 48 | serum | GC-MS | ANOVA, ROC, PCA and PLS-DA analysis | Age, sex, smoking and alcohol consumption | Cis-Aconitic acid, capric acid, caprylic acid, citrate, ethylmalonic acid, fumarate, glutaric acid, glycolic acid, β-hydroxybutyrate, α-hydroxyisocaproic acid, isocitrate, α-ketoglutarate, malate, malonic acid, methylmalonic acid, orotic acid, oxalic acid, oxaloacetate, phosphoenol pyruvate, pimelic acid, pyroglutamic acid, pyruvate, sebacic acid, suberic acid, succinate |
| Lu et al., 2019, Metabolites | Case-control study | Chinese | 144 | 144 | serum | LC-MS | t-test, chi-square test and logistic regression | BMI, history of hypertension, smoking status, HDL-cholesterol, and triglycerides | Glutamic acid, asparagine, arginine, aspartic acid |

Abbreviation: GC, gas chromatography; MS, mass spectrometry; LC, liquid chromatography; NMR, nuclear magnetic resonance spectroscopy; UPLC-TQ-MS, ultra-high performance liquid chromatography tandem quadruple mass spectrometry; FIA-MS/MS, flow injection analysis tandem mass spectrometry; FIA-ESI-MS/MS, flow injection electrospray ionization tandem mass spectrometry; UHPLC-MS/MS, high performance liquid chromatography tandem mass spectrometry; UPLC-QTOF-MS, ultra-high-performance liquid chromatography-quadrupole time-of-flight mass spectrometry; LC-FIA-MS, liquid chromatography-flow injection analysis-mass spectrometry; COSY, correlation spectroscopy; CPMG, Car-Purcell-Meiboom-Gill; HSQC and HMQC, heteronuclear and homonuclear single quantum coherence spectroscopy; NMR-COMP, small molecular compounds window based NMR spectroscopy; GC-TOF-MS, gas chromatograph-time-of-flight mass spectrometry; NPLC-TOF/MS, normal phase liquid chromatography coupled with time of flight mass spectrometry; HPLC-QQQ-MS/GC, HPLC coupled triple quadrupole mass spectrometry; GC-LC-FIA-MS/MS, gas chromatography-liquid chromatography-flow injection analysis mass spectrometry/mass spectrometry; LASSO, least absolute shrinkage and selection operator; OPLS-DA, orthogonal partial least squares-discriminant analysis; ROC, receiver operating characteristic; PCA: principal component analysis; ANOVA, analysis of variance; ANCOVA, analysis of covariance; HCA, hierarchical cluster analysis; FDR, false discovery rate; BMI, body mass index; SBP, systolic blood pressure; DBP, diastolic blood pressure; BUN, blood urea nitrogen; HDL, high density lipoprotein; LPC, lysophosphatidylcholine; a, acyl.

**Table S4.** Characteristics of the metabolites studied in fewer than three publications on prediabetic patients.

| **Author-year-Journal** | **Study design** | **Population** | **Cases N** | **Control N** | **Biological sample** | **Detection method** | **Analytical method** | **Covariates** | **Metabolites** |
| --- | --- | --- | --- | --- | --- | --- | --- | --- | --- |
| Cobb et al., 2016, Diabetes Care | 3-year follow-up study | RISC: 19 centers in 13 countries in Europe; DMVhi: Irish; Botnia: the west coast of Finland | RISC: 332; DMVhi: 183; Botnia: 1325 | RISC: 623; DMVhi; 485; Botnia: 1105 | plasma | LC-MS/MS | ROC and multiple logistic regression analyses | Age, sex, and BMI | α-hydroxybutyric acid, α-ketobutyric acid, α-ketoglutaric acid, β-hydroxybutyric acid, 3-hydroxyisobutyric acid, 3-methyl-2-oxobutyric acid, 3-methyl-2-oxopentanoic acid, 4-methyl-2-oxobutyric acid, linoleoyl-glycerophosphocholine, oleic acid |
| Menni et al., 2013, Diabetes | Case-control study | British | 192 | 1897 | plasma | NA | Random intercept logistic regressions analysis | Age, BMI, batch effect and family relatedness | Dimethylarginine, 2-hydroxybutyrate, N-acetylglycine, 3-methyl-2-oxobutyrate, 3-methyl-2-oxovalerate, 4-methyl-2-oxopentanoate, fructose, mannose, 1, 5-anhydroglucitol, glucose, lactate, arabinose, malate, octanoylcarnitine, 15-methylpalmitate, 10-heptadecenoate (17:1n7), adrenate (22:4n6), arachidonate (20:4n6), myristoleate (14:1n5), palmitoleate (16:1n7), pentadecanoate (15:0), 5-dodecenoate (12:1n7), heptanoate (7:0), pelargonate (9:0), palmitoyl sphingomyelin, cholesterol |
| Tulipani et al., 2016, Clin Chim Acta | Case-control study | Spanish (Málaga) | 12 | 19 | serum | LC-MS/MS, FIA-ESI-MS/MS | the R environment, Cytoscape | Age, sex, and BMI | LPC a C16:0, LPC a C17:0, LPC a C18:0, LPC a C18:1, LPC a C18:2, LPE a 18:1, LPE a 18:2, LPE a 18:0, LPE e 18:0, PC aa 38:6, PC ae 34:0, PC ae C34:1, PC ae C34:2, PC ae C34:3, PC ae C36:2, PC ae C36:3, PC ae C38:0, PC ae C38:5, PC ae C38:6, PC ae C40:1, PC ae C40:6, PE aa 28:5, PE aa 36:0, PE aa 38:0, PE aa 38:1, PE aa 40:2, PE aa 40:3, PE ae 34:1, PE ae 34:2, PE ae 34:3, PE ae 36:2, PE ae 36:3, PE ae 38:2, PE ae 38:3, PE ae 38:6, PE ae 40:3, PE ae 40:5, PE ae 40:6, PS aa 38:4 |
| Andersson-Hall et al., 2018, J Diabetes Res | 6-years follow-up study | Women in Gothenburg | 46 | 139 | serum | NMR Spectroscopy | ANOVA and ANCOVA | BMI and age | Carbohydrates: glucose, mannose Others: glycerol, citrate, pyruvate, acetoacetate, 3-hydroxy-isobutyrate |
| Zeng et al., 2010, Metabolomics | Case-control study | Chinese (Changsha) | 34 | 24 | plasma | GC/MS | PCA and PLS-DA | Age | Lactate, 2-ketoisocaproic acid, α-hydroxyisobutyric acid, b-hydroxybutyric acid, glycerol, threonine, pyroglutamic acid, palmitic acid, linoleic acid, oleic acid |
| Wang-sattler et al., 2017, Mol Syst Biol | Cross-sectional study | Augsburg and the surrounding towns and villages | 340 | 866 | serum | LC-FIA-MS | Multivariate logistic regression and linear regression | Age, sex, BMI, PA, alcohol intake, smoking, SBP and HDL | Acetylcarnitine C2, LPC (18:2) |
| Newgard et al., 2009, Cell Metab | Case-control study | African Americans | 74 | 67 | serum | MS and MS/MS | PCA | Age, race and sex | Arginine, histidine, methionine, ornithine, Isovaleryl/2-methylbutyryl (C5), hexanoyl (C6), octenoyl (C8:1), palmitoleic C16:1, stearic C18:0, oleic C18:1, linoleic C18:2, a-linolenic C18:3, arachidonic C20:4 |
| Ni et al., 2015, EBioMedicine | longitudinal study | Chinese (Shanghai) | 50 | 12 | serum | UPLC-QTOF-MS | OPLS-DA, ROC and logistic regression analysis, hierarchical clustering | Age, sex, BMI, HOMA-IR, and fasting glucose | SFA: C8:0, C10:0, C12:0, C14:0, C15:0, C16:0, C17:0, C18:0, C19:0, C20:0, C22:0, C24:0, C14:0 iso, C16:0 iso, C15:0 iso, C17:0 iso, C18:0 iso MUFA, n-3 PUFA, n-6 PUFA |
| Butte et al., 2015, Am J Clin Nutr | Case-control study | Hispanic America children | 450 | 353 | plasma | UPLC-MS and GC-MS | PCA and mixed-effects linear regression | Sex, age and Tanner stage | Aspartate, creatine, pyroglutamine, N-acetylglycine, histidine, 2-methylbutyrylcarnitine, 3-methyl-2-oxobutyrate, α-hydroxyisovalerate, isovalerylcarnitine, lysine, 2-hydroxybutyrate, α-ketobutyrate, 3-(4)-hydroxyphenyl lactate, acisoga, C-glycosyltryptophan, kynurenate, kynurenine, g-glutamylglutamate, g-glutamylleucine, g-glutamylphenylalanine, g-glutamyltyrosine, bradykinin, hexanoylcarnitine (6), stearoylcarnitine (18), oleoylcarnitine (18:1), 2-aminoheptanoate, 2-aminooctanoate, dodecanedioate, tetradecanedioate, 2-hydroxydecanoate |
| Mastrangelo et al., 2016, Int J Obes (Lond) | Case-control study | Spanish | 50 | 50 | serum | LC-MS, GC-MS and capillary electrophoresis-mass spectrometry | OPLS-DA, PCA, univariate and multivariate analyses | BMI, age, fasting glucose and multiple testing | Taurodeoxycholate, glycodeoxycholate, piperidine, free-carnitine, pyroglutamate, C04-Carnitine, 3-hydroxybutyrate, pyruvate |
| Kim et al., 2010, J Proteome Res | Case-control study | South Korean | 30 | 30 | serum | UPLC-QTOF-MS | PCA and PLS-DA | Age | Betaine, D-pipecolic acid, butyryl carnitine (C4), octanoyl carnitine,linoleyl carnitine, Palmityl carnitine, LPC (18:2), LPC (18:1), LPC (20:2), LPC (18:0), polyunsaturated ω-3, polyunsaturated ω-6 |
| Lee et al., 2015, Obes Res Clin Pract | Cohort study | South Korean | 64 | 45 | plasma | LC-MS/MS and FIA-MS/MS | ANOVA, logistic regression and ROC curve analyses | Age, BMI and waist circumference | Lysine, acylcarnitines (C5) |

Abbreviation: RISC, Relationship Between Insulin Sensitivity and Cardiovascular Disease; DMVhi, Diabetes Mellitus and Vascular Health Initiative; LC-MS/MS, liquid chromatography coupled to tandem mass spectrometry; FIA-ESI-MS/MS, flow injection electrospray ionization tandem mass spectrometry; NMR, nuclear magnetic resonance spectroscopy; GC/MS, gas chromatography/mass spectrometry; LC-FIA-MS, liquid chromatography and flow injection analysis-mass spectrometry; UPLC-QTOF-MS, ultra-performance liquid chromatograph coupled to quadruple time-of-flight mass spectrometry; ROC, receiver operating characteristic; ANOVA, analysis of variance; ANCOVA, analysis of covariance; PCA, principal component analysis; PLS-DA, partial least squares-discriminant analysis; OPLS-DA, orthogonal partial least squares-discriminant analysis; BMI, body mass index; LPC, lysophosphatidylcholine; a, acyl.

**Table S5.** Heterogeneity test and test for overall effect on the forest plot for type 2 diabetes mellitus.

| **Subgroup** | **Studies** | **Participants** | **Statistical Method** | **Effect Estimate** | ***I*^2^ (%)** | **Heterogeneity test** | | **Test for overall effect** | |
| --- | --- | --- | --- | --- | --- | --- | --- | --- | --- |
|  |  |  |  |  |  | **Q** | ***P*** | ***Z*** | ***P*** |
| Valine | 7 | 1252 | Std. Mean Difference (IV, Random, 95% CI) | 0.91 [0.59, 1.23] | 85 | 39.47 | < 0.00001 | 5.53 | < 0.00001 |
| Leucine | 7 | 1252 | Std. Mean Difference (IV, Random, 95% CI) | 0.93 [0.57, 1.29] | 88 | 49.47 | < 0.00001 | 5.05 | < 0.00001 |
| Isoleucine | 7 | 1252 | Std. Mean Difference (IV, Random, 95% CI) | 0.93 [0.60, 1.27] | 86 | 42.24 | < 0.00001 | 5.48 | < 0.00001 |
| Phenylalanine | 6 | 3871 | Std. Mean Difference (IV, Random, 95% CI) | 0.86 [0.42, 1.31] | 94 | 81.05 | < 0.00001 | 3.83 | 0.0001 |
| Glycine | 4 | 3509 | Std. Mean Difference (IV, Fixed, 95% CI) | -0.42 [-0.49, -0.34] | 29 | 4.21 | 0.24 | 10.88 | < 0.00001 |
| Proline | 4 | 608 | Std. Mean Difference (IV, Random, 95% CI) | 0.50 [0.18, 0.82] | 70 | 9.92 | 0.02 | 3.10 | 0.002 |
| Tyrosine | 4 | 496 | Std. Mean Difference (IV, Fixed, 95% CI) | 0.56 [0.37, 0.75] | 43 | 5.22 | 0.16 | 5.82 | < 0.00001 |
| Alanine | 3 | 315 | Std. Mean Difference (IV, Random, 95% CI) | 0.08 [-0.70, 0.86] | 91 | 22.62 | < 0.0001 | 0.20 | 0.84 |
| Glutamine | 3 | 462 | Std. Mean Difference (IV, Random, 95% CI) | 0.58 [-0.32, 1.49] | 94 | 35.09 | < 0.00001 | 1.27 | 0.21 |
| Lysine | 3 | 462 | Std. Mean Difference (IV, Random, 95% CI) | 0.84 [0.28, 1.40] | 85 | 13.32 | 0.001 | 2.96 | 0.003 |
| Glutamate | 3 | 246 | Std. Mean Difference (IV, Random, 95% CI) | 0.63 [0.19, 1.07] | 57 | 4.66 | 0.10 | 2.79 | 0.005 |
| Histidine | 3 | 539 | Std. Mean Difference (IV, Random, 95% CI) | 0.18 [-0.48, 0.83] | 92 | 26.17 | < 0.00001 | 0.53 | 0.60 |
| Serine | 3 | 336 | Std. Mean Difference (IV, Random, 95% CI) | -0.49 [-1.30, 0.32] | 92 | 25.88 | < 0.00001 | 1.18 | 0.24 |

Abbreviations: CI, confidence interval; *I*^2^, I-squared statistic, the proportion of heterogeneity.

**Table S6.** Heterogeneity test and test for overall effect on the forest plot for prediabetes.

| **Subgroup** | **Studies** | **Participants** | **Statistical Method** | **Effect Estimate** | ***I*^2^ (%)** | **Heterogeneity test** | | **Test for overall effect** | |
| --- | --- | --- | --- | --- | --- | --- | --- | --- | --- |
|  |  |  |  |  |  | **Q** | ***P*** | ***Z*** | ***P*** |
| Leucine | 10 | 2058 | Std. Mean Difference (IV, Random, 95% CI) | 1.07 [0.61, 1.54] | 95 | 179.96 | < 0.00001 | 4.52 | < 0.00001 |
| Valine | 9 | 2000 | Std. Mean Difference (IV, Random, 95% CI) | 1.29 [0.75, 1.83] | 96 | 202.78 | < 0.00001 | 4.68 | < 0.00001 |
| Isoleucine | 9 | 1998 | Std. Mean Difference (IV, Fixed, 95% CI) | 0.45 [0.36, 0.54] | 48 | 15.26 | 0.05 | 9.83 | < 0.00001 |
| Tyrosine | 8 | 1542 | Std. Mean Difference (IV, Random, 95% CI) | 1.10 [0.58, 1.62] | 94 | 114.96 | < 0.00001 | 4.12 | < 0.0001 |
| Phenylalanine | 7 | 1482 | Std. Mean Difference (IV, Random, 95% CI) | 0.92 [0.40, 1.43] | 94 | 92.75 | < 0.00001 | 3.51 | 0.0004 |
| Propionylcarnitine (C3) | 7 | 1354 | Std. Mean Difference (IV, Random, 95% CI) | 1.65 [0.83, 2.48] | 97 | 189.00 | < 0.00001 | 3.93 | < 0.0001 |
| Glycine | 6 | 1352 | Std. Mean Difference (IV, Random, 95% CI) | -0.76 [-1.00, -0.51] | 65 | 14.19 | 0.01 | 3.13 | < 0.00001 |
| Alanine | 6 | 1302 | Std. Mean Difference (IV, Random, 95% CI) | 0.57 [0.30, 0.83] | 73 | 18.33 | 0.003 | 4.17 | < 0.0001 |
| Proline | 5 | 499 | Std. Mean Difference (IV, Fixed, 95% CI) | 0.41 [0.23, 0.59] | 3 | 4.11 | 0.39 | 4.50 | < 0.00001 |
| Glutamate | 5 | 1172 | Std. Mean Difference (IV, Random, 95% CI) | 0.61 [0.20, 1.02] | 85 | 27.02 | < 0.0001 | 2.91 | 0.004 |
| Serine | 5 | 1202 | Std. Mean Difference (IV, Random, 95% CI) | -0.37 [-0.70, -0.04] | 81 | 20.53 | 0.0004 | 2.18 | 0.03 |
| Carnitine (C0) | 4 | 1028 | Std. Mean Difference (IV, Random, 95% CI) | -0.25 [-1.42, 0.93] | 97 | 85.79 | < 0.00001 | 0.41 | 0.68 |
| Citrulline | 4 | 1144 | Std. Mean Difference (IV, Fixed, 95% CI) | -0.37 [-0.49, -0.25] | 0 | 1.07 | 0.78 | 6.13 | < 0.00001 |
| Asparagine | 4 | 1144 | Std. Mean Difference (IV, Random, 95% CI) | -0.16 [-0.78, 0.46] | 94 | 50.56 | < 0.00001 | 0.51 | 0.61 |
| Tryptophan | 3 | 963 | Std. Mean Difference (IV, Random, 95% CI) | 2.44 [-0.12, 4.99] | 99 | 1450.50 | < 0.00001 | 1.87 | 0.06 |
| Myristate (C14:0) | 3 | 263 | Std. Mean Difference (IV, Random, 95% CI) | -0.38 [-1.86, 1.10] | 96 | 49.63 | < 0.00001 | 0.50 | 0.61 |
| Palmitic acid (C16:0) | 3 | 263 | Std. Mean Difference (IV, Random, 95% CI) | 0.85 [0.44, 1.26] | 51 | 4.04 | 0.13 | 4.06 | < 0.0001 |
| 2-aminoadipic acid | 3 | 250 | Std. Mean Difference (IV, Fixed, 95% CI) | 0.69 [0.43, 0.95] | 0 | 0.99 | 0.61 | 5.24 | < 0.00001 |
| Lysine | 3 | 1003 | Std. Mean Difference (IV, Fixed, 95% CI) | 0.36 [0.24, 0.49] | 0 | 1.54 | 0.46 | 5.66 | < 0.00001 |

Abbreviations: CI, confidence interval; *I*^2^, I-squared statistic, the proportion of heterogeneity.
